# Large‐Sized Poly (Triazine Imide) Crystals with Minimized Defects for High‐Efficiency Overall Water Splitting

**DOI:** 10.1002/advs.202510084

**Published:** 2025-07-29

**Authors:** Chong Wang, Na Shi, Yulin Zhou, Yichun Lu, Jingru Zhuang, Hongwu Liao, Chengning Ye, Hanhui Lei, Xiangfeng Lin, Jiaxian Zheng, Terence Xiaoteng Liu, Zhanhui Yuan

**Affiliations:** ^1^ College of Materials Engineering Fujian Agriculture and Forestry University Fuzhou 350002 China; ^2^ Department of Chemistry The Chinese University of Hong Kong Shatin Hong Kong SAR 999077 China; ^3^ State Key Laboratory of Marine Pollution City University of Hong Kong Kowloon Tong Hong Kong SAR 999077 China; ^4^ Department of Mechanical and Construction Engineering Northumbria University Newcastle‐upon‐Tyne NE1 8ST U.K.

**Keywords:** poly triazine imide, photocatalysis, water splitting, particle size, crystallinity

## Abstract

Poly (triazine imide) (PTI), a highly crystalline carbon nitride, has attracted considerable attention due to its capacity to achieve one‐step‐excitation overall water splitting. The crystallinity of PTI crystals can be substantially enhanced by manipulating the polymerization temperature and the type of molten salt. However, the particle size of synthetic crystals is usually less than 200 nm, and the synthesis of larger and well‐defined PTI crystals has yet to be accomplished. In this study, a novel precursor engineering strategy is implemented utilizing melon‐type carbon nitride as the architectural template, coupled with LiCl/KCl eutectic molten salt system, to orchestrate the crystallization of PTI under high‐temperature/high‐pressure conditions. Comparative analysis reveals that the PTI synthesized through this methodology exhibits marked crystallographic superiority over conventional dicyandiamide‐derived counterparts, manifesting as notably reduced lattice imperfections and an extended π‐conjugated network. These structural enhancements culminate in the formation of faceted single‐crystalline domains with characteristic dimensions exceeding 500 nm. The optimized single‐crystal PTI showcases a high apparent quantum efficiency of 13.1% (λ = 365 nm) in overall water splitting for hydrogen production. This work establishes the critical role of precursor architectural compatibility in governing crystalline perfection and functional performance in carbon nitride‐based photocatalytic systems.

## Graphical abstract

Large‐sized PTI single crystals with significantly reduced defects and an extended π‐conjugated network are successfully constructed by using melon as a precursor. The optimized PTI achieves a high apparent quantum efficiency of 13.1% @365 nm for overall water splitting. This work highlights the critical role of precursor architectural compatibility in enhancing crystallinity and photocatalytic performance in carbon nitride materials.

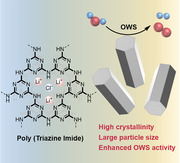

## 1 Introduction

Within the framework of global carbon neutrality initiatives, hydrogen has emerged as a preeminent candidate for sustainable energy vectors due to its zero‐emission characteristics and renewable nature.^[^ ^1^ ^]^ Conventional hydrogen production methodologies, predominantly reliant on steam methane reforming and coal gasification, inherently suffer from substantial carbon emissions and the unsustainable exploitation of finite petrochemical reserves.^[^ ^2^, ^3^, ^4^ ^]^ Conversely, photocatalytic overall water splitting (OWS) for hydrogen production involves no carbon footprint and utilizes solar energy, thereby establishing an environmentally benign and energetically sustainable paradigm.^[^ ^5^ ^]^ The photocatalytic water splitting system can be categorized into one‐step excitation and two‐step excitation processes, among which the one‐step excitation is considered a straightforward and scalable route for solar‐to‐hydrogen (STH) conversion.^[^ ^6^, ^7^ ^]^ For instance, Domen et al. accomplished OWS‐based hydrogen generation on a 100 m^2^ panel system by employing Al‐doped SrTiO_3_ particulate photocatalysts.^[^ ^8^ ^]^ This milestone achievement effectively demonstrated the practical viability of OWS when implemented on a large scale, providing a solid basis for further research and development in the relevant domain.

Photocatalyst plays a pivotal role in the one‐step excitation overall water splitting reaction.^[^ ^9^ ^]^ To date, the utilization of inorganic semiconductors as OWS photocatalysts has been predominantly driven by their inherent stability and specific activity.^[^ ^10^ ^]^ In the domain of photocatalytic water splitting, a significant shift is underway: the transition from conventional inorganic photocatalysts to organic alternatives is gradually becoming more prominent.^[^ ^11^, ^12^ ^]^ The molecular structure of organic semiconductors is designable, and the band structure, light absorption range, and charge transfer characteristics can be finely controlled through precise chemical synthesis to meet the complex reaction requirements of OWS under different conditions.^[^ ^13^, ^14^ ^]^ Furthermore, certain organic photocatalysts demonstrate exceptional solubility and processability, facilitating the synthesis of photocatalytic materials in diverse forms, such as films and nanofibers.^[^ ^15^, ^16^ ^]^ This adaptability to various reaction devices and the concomitant reduction in large‐scale preparation costs signify a promising new avenue for the industrial implementation of photocatalytic overall water splitting technology.

**Scheme 1:**
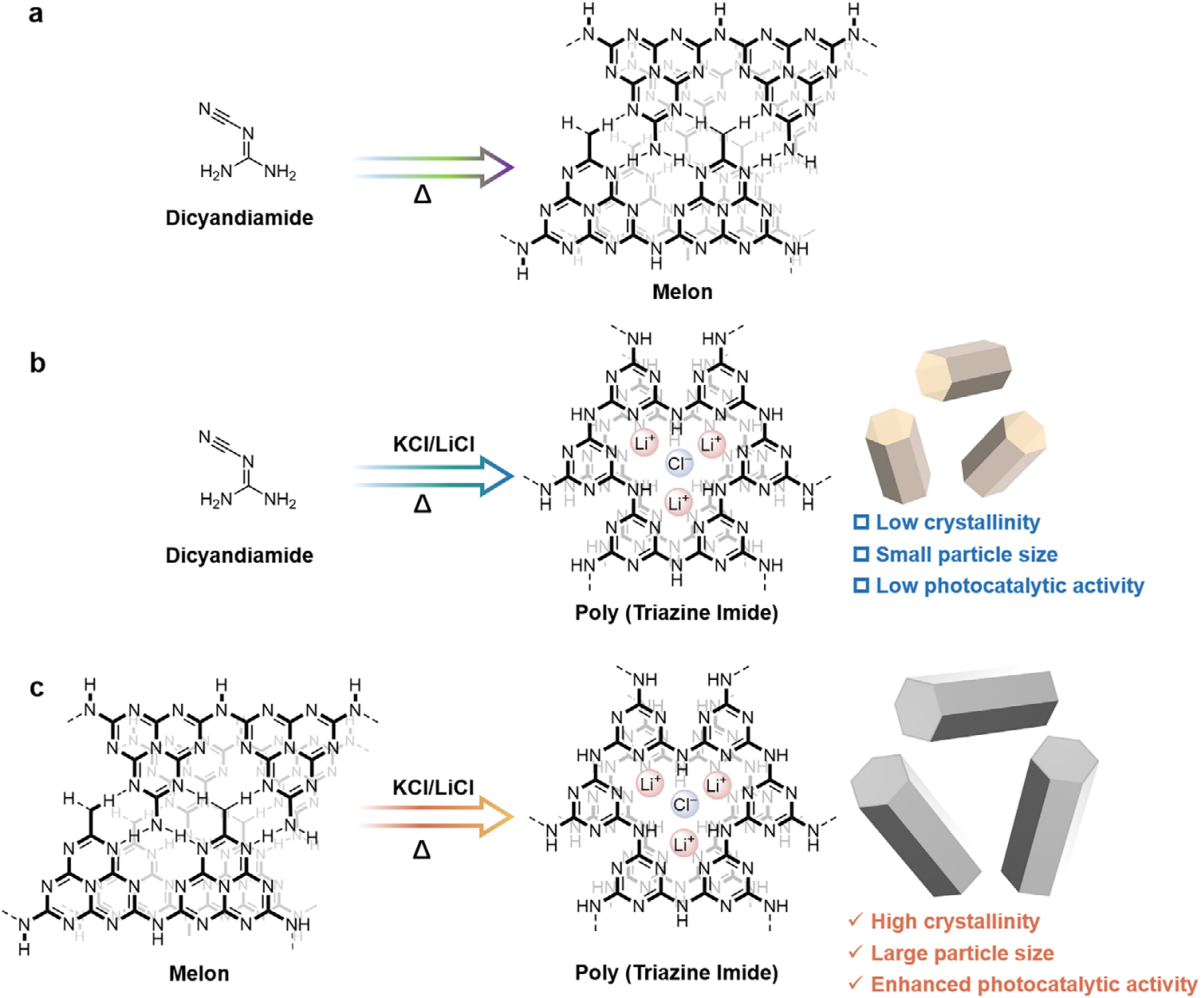
Diagram of the synthesis of different types of carbon nitride.

As a typical representative of covalent polymers, polymeric carbon nitride (PCN) has garnered significant attention in photocatalytic water splitting due to its distinctive properties.^[^ ^17^ ^]^ The construction of PCN is predicated on two fundamental building blocks: triazine (C_3_N_3_) and heptazine unit (C_6_N_7_).^[^ ^18^, ^19^, ^20^, ^21^ ^]^ The simplest and most studied PCN is melon, which can be synthesized by thermal polymerization from nitrogen‐rich precursors such as dicyandiamide (**Scheme** **1**a). However, the connection mode of melon results in an amorphous structure with significant electron‐hole recombination, which leads to a low efficiency of one‐step excitation OWS. Many modification approaches have been employed to improve the OWS efficiency of PCN, among which molten salt‐assisted synthesis is regarded as the most efficacious strategy.^[^ ^22^ ^]^ For instance, PCN prepared by thermal polymerization of nitrogen‐rich precursors in binary molten salts exhibits a poly (triazine imide) (PTI) structure and has been successfully applied in OWS (Scheme 1b).^[^ ^23^ ^]^ In‐depth studies have demonstrated that, while PTI is classified as a crystalline carbon nitride, its structure exhibits numerous structural defects. These defects have been identified as potential centers for electron‐hole recombination. Consequently, optimizing the synthesis process to yield highly crystalline PTI is challenging and imperative.

Despite the challenges associated with polymerization control at elevated temperatures, methodologies can be employed to enhance the conjugation and crystallinity of PTI structures. For instance, the melting point of the binary molten salt NaCl/LiCl is 552 °C, which is closer to the temperature of precursor polymerization than the melting point of KCl/LiCl (353 °C) and has a superior solvation effect. The prepared PTI has fewer structural defects and an expanded conjugated system.^[^ ^24^ ^]^ Similarly, the molten salt selection can be extended to three components (KCl/NaCl/LiCl).^[^ ^25^ ^]^ Furthermore, the judicious selection of the appropriate type of precursor has been demonstrated to be an effective method of expanding the PTI conjugated system.^[^ ^26^ ^]^ Very recently, Zhang et al. discovered that utilizing binary precursors (melamine/cyanuric acid) facilitates the generation of intermediates, thereby enhancing the polymerization reaction.^[^ ^27^ ^]^ Notably, the particle size of most existing PTI single crystals is typically less than 200 nm, a consequence of the complexity of the thermal polymerization reaction. While the larger single crystal size does not necessarily mean better OWS efficiency, it corresponds to a more extensive π‐conjugated system and a reduced number of edge structural defects, which are advantageous for the transmission of carriers. Inspired by state‐of‐the‐art inorganic material synthesis methods, increasing the calcination time may be feasible to synthesize large‐sized PTI single crystals.^[^ ^28^ ^]^ Unfortunately, increasing the calcination time can improve the PTI single crystal size to a certain extent, but it will also cause the decomposition of the polymer structure and produce defects. Consequently, developing a simultaneously facile and scalable methodology for synthesizing defect‐minimized PTI single crystals with micron‐scale dimensions and enhanced OWS activity remains a persistent challenge in advanced photocatalyst engineering.

Presently, synthesizing highly crystalline PTI requires a vacuum‐tight system to establish a high‐pressure environment.^[^ ^29^ ^]^ While the precise mechanism by which pressure influences the formation of PTI crystals remains to be elucidated, the requirement for high pressure is attributable to the fact that PTI synthesized under ambient pressure invariably adopts an amorphous state.^[^ ^30^ ^]^ However, current synthesis methods often involve pre‐calcination of the precursor with the salt at a lower temperature than the polymerization temperature to release the ammonia produced by the depolymerization process.^[^ ^31^ ^]^ This strategy can mitigate excessive pressure within the reactor; however, it is incongruent with the requisite high‐pressure conditions, thereby hindering the synthesis of large‐sized PTI single crystals. In this study, we sought to optimize the synthesis process by replacing dicyandiamide with melon‐based PCN as a precursor and omitting the pre‐calcination process to prepare PTI single crystals (Scheme 1c). Benefiting from the expansion of the conjugated system, the resulting PTI single crystals become larger, and the carrier transport efficiency is enhanced. The synthesis method that has been developed can shorten the polymerization reaction time and effectively avoid the structural defects that may be caused by long‐term calcination. Upon loading platinum and cobalt oxides as cocatalysts, stoichiometric amounts of hydrogen and oxygen were simultaneously produced on well‐defined PTI single crystals. These results are of great significance because they provide a method for growing large PTI crystal particles and offer new ideas for the large‐scale preparation of PTI materials.

## 2 Results and Discussion

**Figure 1:**
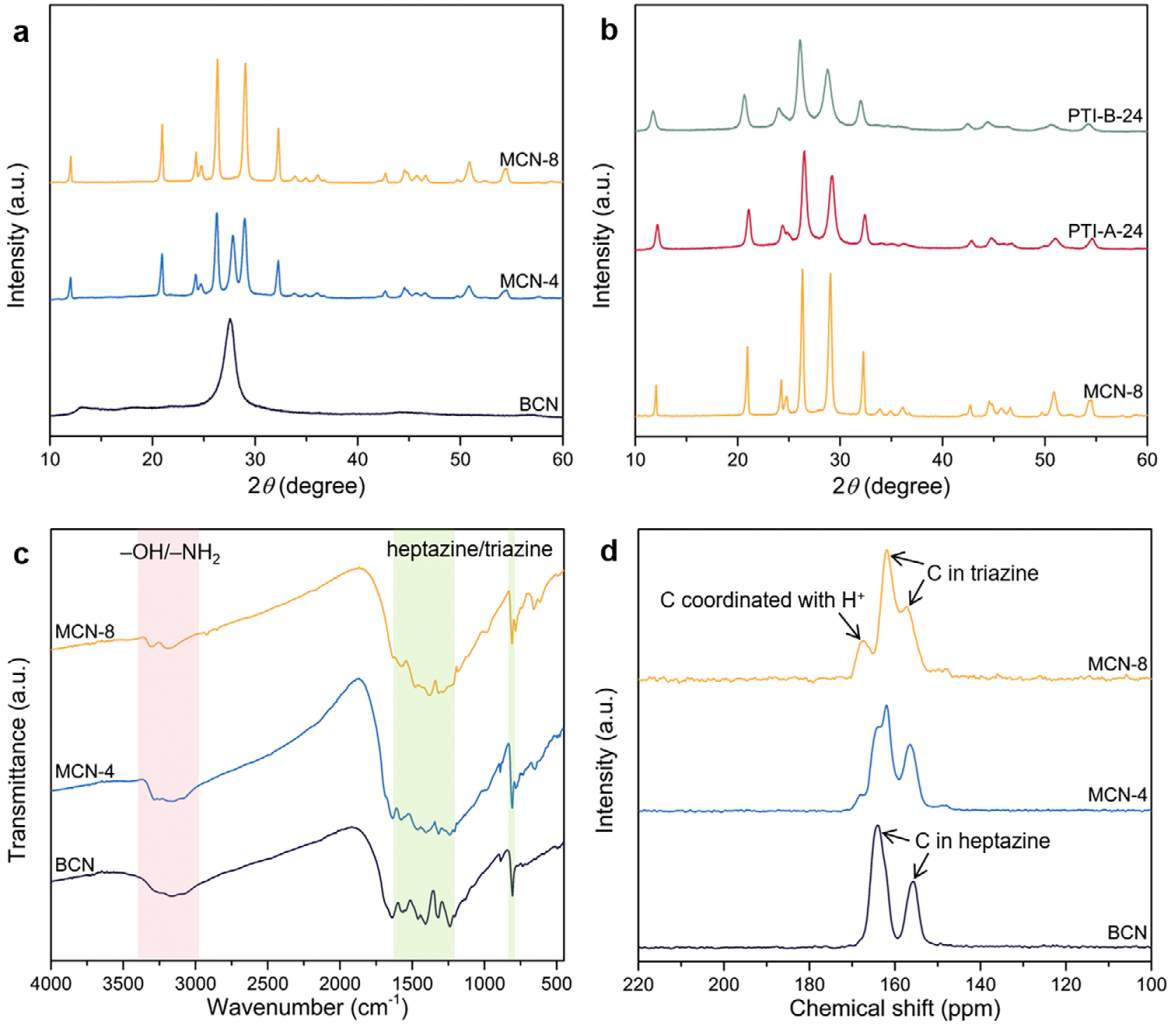
a,b) The XRD patterns of the samples. c) The FTIR spectra and d) Solid‐state ^13^C CP‐MAS NMR spectra of the samples.

In a typical experimental procedure, bulk carbon nitride (BCN) was synthesized via thermal polycondensation of dicyandiamide under an inert atmosphere. Subsequently, the as‐prepared BCN underwent molten salt‐assisted calcination (KCl/LiCl eutectic mixture) at elevated temperatures to generate a series of modified carbon nitride materials denoted as MCN‐X, where X corresponds to the calcination duration (4 and 8 h). The phase diagram of the eutectic point of the KCl/LiCl binary molten salt system is shown in Figure S1 (Supporting Information). The complete synthesis protocol, including temperature profiles and precursor ratios, is comprehensively outlined in the Supporting Information. X‐ray diffraction (XRD) was initially conducted to investigate the structural divergence among synthesized samples. As demonstrated in **Figure** **1**a, BCN exhibits two characteristic diffraction peaks of melon (2θ = 13.2 and 27.6°), which are indexed to the (100) in‐plane structural periodicity and the (002) interlayer *π*–*π* stacking interactions of the heptazine units.^[^ ^32^ ^]^ As the duration of molten salt treatment increased, the diffraction peaks of melon undergo a gradual weakening and eventual disappearance. Concurrently, the diffraction peaks of the PTI structure exhibit a substantial enhancement. This phenomenon manifests that the depolymerization of the heptazine units of melon into triazine units occurs in the presence of molten salt, followed by polymerization to form the PTI structure. It is worth noting that the diffraction peaks of melon and PTI appear simultaneously in the pattern of MCN‐4, which means the formation of an intramolecular heterojunction.

Furthermore, a comparative analysis of MCN‐8 with PTI‐A‐24 (melon as the precursor, two‐step calcination) and PTI‐B‐24 (dicyandiamide as the precursor, two‐step calcination) was conducted, as reported in the extant literature (Figure 1b). The XRD peaks of MCN‐8 exhibit greater sharpness and reduced full width at half‐maximum (FWHM), suggesting that the proposed method can yield PTI single crystals with enhanced crystallinity while concomitantly reducing the synthesis time (Table S1, Supporting Information). Similarly, the observed enhancement in diffraction peak intensity correlates with increased crystallite dimensions. For PTI‐type polymers, the length of calcination time has a direct impact on crystallinity. When the molten salt treatment was reduced to 8 h, a significant decrease in the XRD diffraction peak intensity of PTI‐A and PTI‐B was observed (Figure S2, Supporting Information). For the MCN samples, there is minimal variation in the diffraction peak intensity between MCN‐8 and MCN‐24. This phenomenon also demonstrates that our method can effectively shorten the preparation time of high‐crystalline PTI. In the absence of elevated pressure, preparing high‐crystalline PTI‐based carbon nitride is unfeasible, irrespective of dicyandiamide or melon as a precursor in the molten salt process (Figure S3, Supporting Information). The structure prepared using melon as the precursor exhibits greater similarity to potassium poly (heptazine imide) (K‐PHI).^[^ ^33^, ^34^, ^35^ ^]^ This outcome substantiates the indispensability of a high‐pressure environment within a closed system for synthesizing high‐crystalline PTI. However, the mechanism of high‐temperature polymerization is complex, and its observation and control are challenging, particularly under sealed conditions. Therefore, the effect of pressure on PTI crystal growth kinetics is still unclear and will be a focus of future research.

The chemical structure of the samples was further confirmed by Fourier transform infrared (FT‐IR) spectroscopy. The FT‐IR spectra in Figure 1c reveal a characteristic vibrational mode centered at 810 cm^−1^ and a broad absorption envelope spanning 1200–1700 cm^−1^ across all samples, corresponding to the out‐of‐plane breathing mode and in‐plane stretching vibrations of the carbon‐nitrogen heterocyclic framework, respectively.^[^ ^36^ ^]^ While the overall spectra of the three samples are relatively similar, some differences can still be observed. The FT‐IR spectrum of BCN displays an extensive band ≈3200–3500 cm^−1^, corresponding to the presence of uncondensed amino groups (─NH_x_) and adsorbed water molecules on the melon surface. Benefiting from the more regular linking mode and lower hydrogen content of the PTI structure (Table S2, Supporting Information), the ─NH_x_ peak intensity of the MCN samples gradually weakened. Furthermore, the structural transformation process from melon to PTI was corroborated by solid‐state ^13^C CP‐MAS NMR measurements. As demonstrated in Figure 1d, two typical signals (163.9 and 155.7 ppm) of carbon atoms in the heptazine ring can be observed in the spectrum of BCN.^[^ ^37^ ^]^ After molten salt treatment, the signal of heptazine in the spectra of MCN samples weakens, and the characteristic signals (161.9 and 157.2 ppm) of carbon atoms in the triazine ring appear.^[^ ^38^ ^]^ Given that the MCN samples possess a full or partial PTI structure, the signal at 168 ppm is ascribed to the C species adjacent to the non‐protonated triazine ring N atom.^[^ ^39^ ^]^ The characteristic signals of triazine and heptazine rings appear simultaneously in MCN‐4, substantiating the existence of an intramolecular heterojunction in the frameworks.

**Figure 2:**
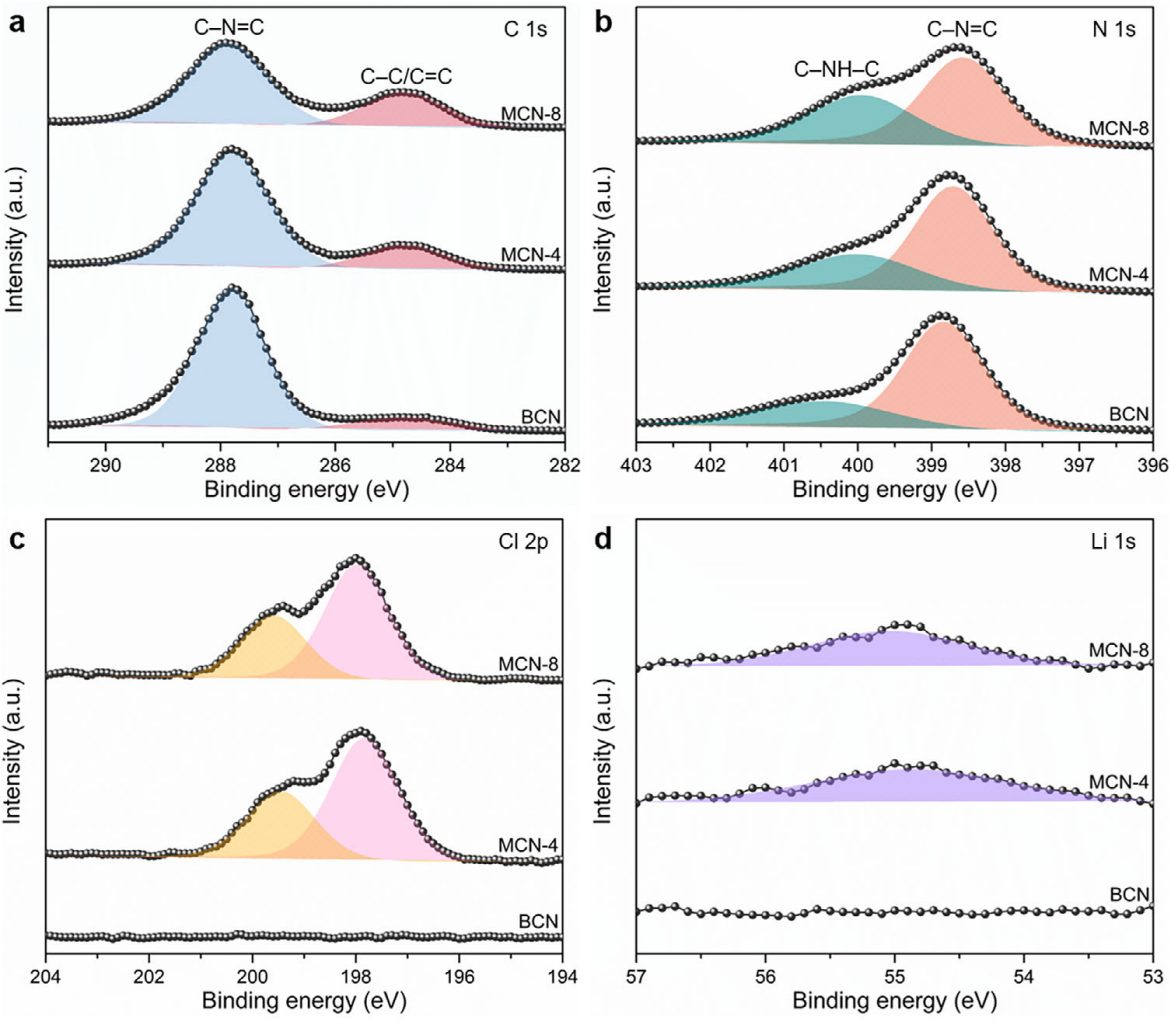
High‐resolution XPS spectra of a) C 1s, b) N 1s, c) Cl 2p, and d) Li 1s for the samples.

X‐ray photoelectron spectroscopy (XPS) was performed to verify the local chemical composition of the catalysts. Given the presence of a CN heterocyclic structure in all three samples, comparable C and N spectra are observed (**Figures** **2**a,b). For MCN‐8, the binding energies of 287.9 and 284.8 eV are attributed to sp^2^‐hybridized carbon (N─C═N) in triazine rings and graphitic carbon (C═C/C─C), respectively.^[^ ^40^ ^]^ The nitrogen spectrum of MCN‐8 can be deconvoluted into two peaks located at 398.6 and 400.0 eV, which are attributed to the N species in the triazine ring (C─N═C) and the bridging amino groups (C─NH─C).^[^ ^41^ ^]^ Since molten salt can incorporate Li^+^ and Cl^–^ into the PTI structure, the Cl 2p and Li 1s signals are detected in MCN‐4 and MCN‐8 (Figures 2c,d). This observation serves as additional evidence indicative of the formation of the PTI structure. Furthermore, the absence of signals corresponding to K 2p (Figure S4, Supporting Information) indicates that the K element is not present in the frameworks of the MCN samples.

**Figure 3:**
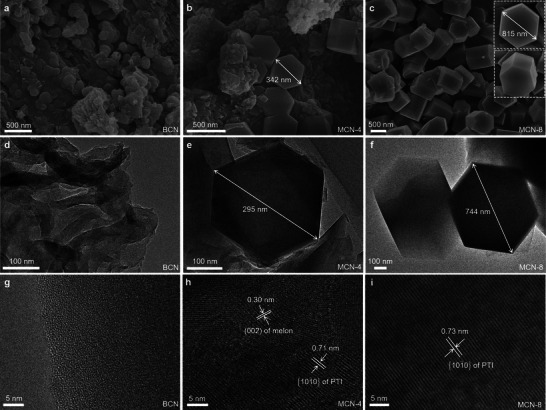
The SEM and TEM of the samples.

The morphological features of the samples were subsequently characterized by scanning electron microscopy (SEM) and transmission electron microscopy (TEM). As shown in **Figures** **3**a,d, BCN presents a structure of irregularly stacked sheets and particles. No lattice fringes are observed in the high‐resolution TEM image of BCN (Figure 3g), thereby confirming its amorphous nature. Upon implementing a four‐hour molten salt treatment, the disordered morphology of MCN‐4 is preserved while simultaneously generating some hexagonal prisms (Figures 3b–e). These hexagonal prisms are characteristic of PTI crystals, comprising two basal planes {0001} with hexagonal facets and six equivalent prismatic facets $\left\{10\bar{1}0\right\}$ oriented along the c‐axis.^[^ ^42^ ^]^ In MCN‐4, the average length of the hexagonal prisms on the {0001} plane ranges from 270 to 360 nm. In Figure 3h, two distinct lattice spacings of 0.30 and 0.71 nm are evident, corresponding to the (002) crystal plane of melon and the $\;\{10\bar{1}0\}$ crystal plane of PTI, respectively. Prolonged molten salt‐assisted thermal annealing induces a thermodynamically driven morphological evolution, resulting in the predominance of the hexagonal prism morphology within the MCN‐8 sample (Figure 3c; Figure S5, Supporting Information). Notably, the size of the hexagonal prisms in MCN‐8 exhibited a further augmentation, reaching 560 to 860 nm (Figure 3c insert, 3f; Figure S6, Supporting Information). Despite an extended calcination time of 24 h, the large‐sized hexagonal prism morphology is retained (Figure S7, Supporting Information). Likewise, the distinct $\left\{10\bar{1}0\right\}$ lattice spacings are observed in MCN‐8, demonstrating its high crystallinity (Figure 3i; Figure S8, Supporting Information). Furthermore, the morphology and particle size of PTI‐A and PTI‐B are also investigated. When the molten salt treatment time is adjusted to 8 h, the PTI‐A and PTI‐B show irregular accumulation of small particles without evident hexagonal prism formation (Figure S9, Supporting Information). Extending the calcination time (24 h) has been demonstrated to yield hexagonal prism crystals (Figures S10 and S11, Supporting Information); however, these crystals exhibit a substantially smaller particle size than that of MCN‐8. Based on the aforementioned analysis, it can be concluded that the method under investigation can synthesize PTI single crystals with high crystallinity and large size while reducing calcination (Scheme S1, Supporting Information). As demonstrated in Figure S12 (Supporting Information), the BET specific surface area (S_BET_) of BCN, MCN‐4, and MCN‐8 are 15, 82, and 11 m^2^·g^−1^, respectively. The heptazine rings in melon first depolymerize to form triazine rings under the action of molten salt, and then polymerize to form the PTI structure. This depolymerization‐repolymerization synthesis has the potential to modify the structure of the sample, which may account for the observation that MCN‐4 exhibits the highest S_BET_.

**Figure 4:**
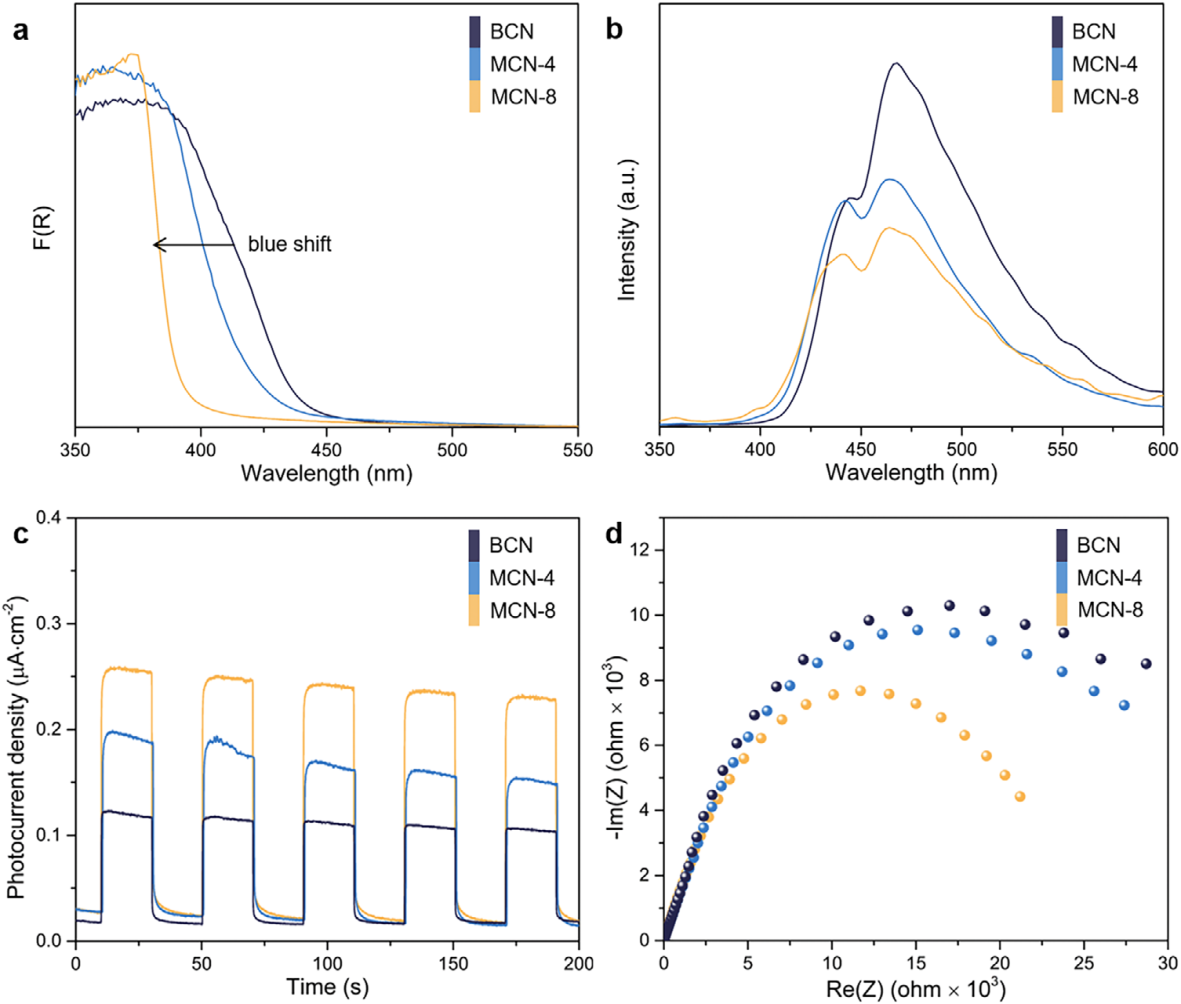
Properties measurement of the photocatalyst. a) UV–vis DRS. b) PL spectra under 325 nm excitation. c) Photocurrent (λ > 300 nm). d) Nyquist plots.

The changes in the light absorption of the catalysts, resulting from the structural transformation, were characterized by UV–vis diffuse reflectance spectroscopy (DRS). Compared with BCN, the light absorption edge of the MCN sample undergoes a significant blue shift (**Figure** **4**a), and the color of the sample changes from light yellow to grayish white (Figure S13, Supporting Information). An increase in the synthesis temperature from 550 to 600 °C results in the emergence of a novel absorption peak within the 400–500 nm range. This phenomenon can be attributed to the n→π^*^ transition, which arises from the presence of N defects within the structural framework (Figure S14, Supporting Information). The band gap energies of BCN, MCN‐4, and MCN‐8 are calculated to be 2.72, 2.81, and 3.11 eV, respectively, based on the Tauc plots (Figure S15, Supporting Information). This blue‐shift trend is also observed in the photoluminescence (PL) spectra, indicating that the band gap of PTI is broader than that of melon (Figure 4b). MCN‐8 exhibits the lowest PL intensity, implying that the recombination of photogenerated charges is significantly suppressed. Figure S16 (Supporting Information) displays the PL spectra under 420 nm excitation. Compared with BCN, the PL emission peak of the MCN samples is significantly weakened and blue‐shifted. Among them, the PL intensity of MCN‐8 is the weakest, which may be attributed to its lowest light absorption intensity at 420 nm. Time‐resolved photoluminescence (TRPL) spectroscopy was performed to elucidate the charge carrier dynamics. As shown in Figure S17 (Supporting Information), the fitted average carrier lifetime of MCN‐8 is 4.0 ns, shorter than that of BCN (9.6 ns). This change indicates that the improvement of crystallinity and conjugated system enables the rapid transmission of carriers, allowing them to participate in the photocatalytic reaction more effectively, rather than staying on the catalyst surface.

The electron transport properties were investigated through time‐resolved photocurrent response measurements and electrochemical impedance spectroscopy (EIS). As shown in Figures 4c,d, MCN‐8 demonstrates a superior photocurrent under 300 W Xe lamp irradiation (λ > 300 nm), coupled with the lowest charge‐transfer resistance derived from Nyquist plot analysis, suggesting that the formation of the PTI structure facilitates interfacial charge separation efficiency. Subsequently, the flat‐band potentials (*E_fb_*) of the synthesized materials were ascertained via Mott‐Schottky analysis. The *E_fb_* of BCN, MCN‐4, and MCN‐8 were determined to be −0.75, −0.53, and −0.46 V versus normal hydrogen electrode (NHE), respectively (Figure S18, Supporting Information). It can be concluded that the approximate conduction band potentials (*E_C_*) are −0.85 V for BCN, −0.63 V for MCN‐4, and −0.56 V for MCN‐8. Combined with the bandgap value, the corresponding valence band potentials (*E_V_*) of the samples are calculated. As illustrated in Figure S19 (Supporting Information), the energy band positions of the three samples demonstrate the thermodynamic feasibility of overall water splitting. For the OWS reaction, the water oxidation reaction (OER) involves the transfer of four electrons (2H_2_O + 4h^+^ → O_2_ + 4H^+^), and the reaction kinetics are slow. Compared with the original BCN, the *E_V_* of the MCN sample shifts toward a more positive potential. A more positive *E_V_* is associated with a more vigorous water oxidation capacity, which is advantageous for enhancing the whole efficiency of OWS.^[^ ^43^ ^]^

**Figure 5:**
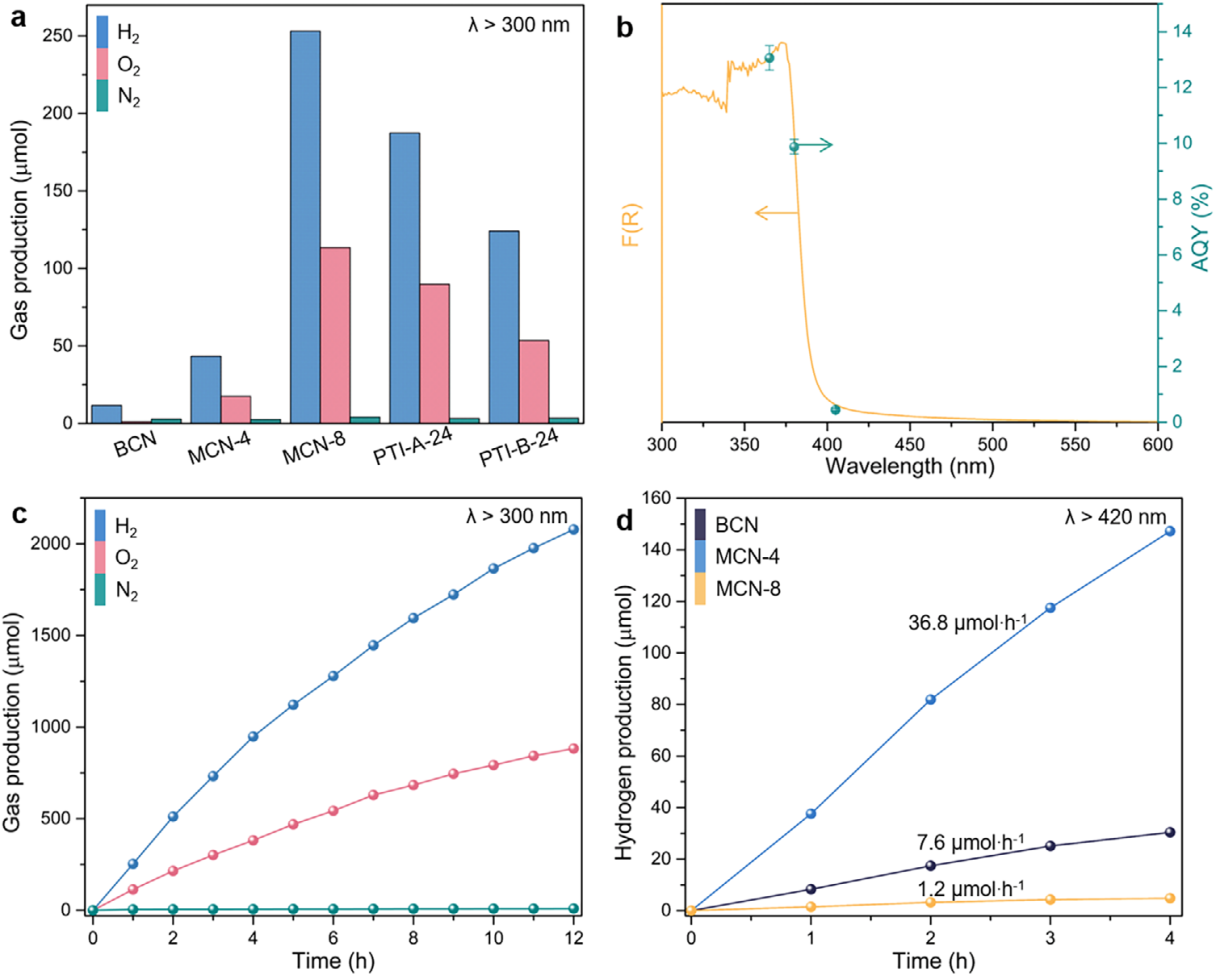
a) The photocatalytic OWS activity of the samples (300 W Xe lamp, λ > 300 nm, 1 h). b) Wavelength‐dependent AQY of MCN‐8 (right axis) and UV/Vis absorption spectrum of MCN‐8 (left axis). c) Time course of OWS by MCN‐8 catalysts. d) Photocatalytic hydrogen production over BCN and MCN samples under visible light irradiation (λ > 420 nm).

Integrating the comprehensive characterization data, it is reasonable to anticipate that the MCN samples are promising catalysts in OWS reactions. Pt and Co are loaded on the material surface as hydrogen production and oxygen production co‐catalysts, respectively. The co‐catalyst content is determined by inductively coupled plasma‐optical emission spectrometer (ICP‐OES) (Table S3, Supporting Information). As shown in **Figure** **5**a, BCN could only catalyze a low H_2_ production of 11.6 µmol after 1 h‐irradiation (λ > 300 nm). It is noteworthy that only 1.1 µmol of O_2_ was detected, which may be attributable to the fact that the valence band position of BCN is not sufficiently positive, thereby resulting in inadequate water oxidation ability. Conversely, the generation of the PTI structure enables the MCN samples to achieve OWS with a stoichiometric ratio of H_2_ and O_2_ approaching 2:1. Notably, MCN‐8 demonstrates the best OWS efficiency, achieving H_2_ and O_2_ production of 253.1 and 113.4 µmol, respectively. The OWS activity of MCN‐8 is also significantly higher than that of PTI‐A‐24 and PTI‐B‐24, which verifies that higher crystallinity and larger crystal size will improve the photocatalytic performance. The relationship between the duration of molten salt treatment and photocatalytic activity was then investigated. As the molten salt treatment duration increases from 0 to 8 h, the OWS activity of the MCN samples gradually increases (Figure S20, Supporting Information), which is attributed to the growth of the PTI structure and crystals. An extension of the treatment time to 24 h produces negligible alterations in the OWS activity, and the XRD diffraction peak intensities of MCN‐8, MCN‐12, and MCN‐24 are almost the same (Figure S21, Supporting Information). Increasing the treatment time to 36 h may lead to a decline in the XRD diffraction peak intensity and a widening of the FWHM, reducing the OWS activity. Thus, it can be concluded that OWS efficiency is positively correlated with the crystallinity of PTI. The effect of Li ion incorporation into the PTI structure on the OWS activity was then investigated. Acid treatment can replace the Li ions in MCN‐8 with protons (H^+^), a phenomenon that is well‐documented in the field of ionic carbon nitride. (Figure S22, Supporting Information).^[^ ^44^, ^45^ ^]^ Figure S23 (Supporting Information) illustrates that the OWS activity of the samples remains unchanged before and after the acid treatment, indicating that Li^+^ in the structure is not an influencing factor of OWS.

Subsequently, wavelength‐dependent apparent quantum yield (AQY) measurements were performed. As demonstrated in Figure 5b, AQY is estimated to be 13.1% at 365 nm for the MCN‐8 sample. The AQY values demonstrate a strong correlation with the DRS pattern of MCN‐8, indicating that the OWS is driven by the absorption of incident photons. Given the challenges associated with synthesizing large‐sized and highly crystalline PTI single crystals, an AQY of 13.1% is desirable (Table S4, Supporting Information).^[^ ^46^, ^47^ ^]^ Furthermore, MCN‐8 has been demonstrated to facilitate continuous water splitting for H_2_ and O_2_ production (Figure 5c). It should be noted that the ratio of H_2_ to O_2_ increases beyond 2:1 after a 12‐h reaction period. In addition to the slow kinetics of the four‐electron OER reaction, this phenomenon is also related to the competition between the two‐electron OER (2H_2​_O + 2h^+^ → H_2_O_2_​ + 2H^+^). To verify the hypothesis, titanium sulfate colorimetry was employed to detect the generation of H_2_O_2_ in the reaction solution. A characteristic absorption peak of the Ti^IV^‐H_2_O_2_ complex appeared ≈407 nm, confirming the occurrence of two‐electron OER (Figure S24, Supporting Information). Figure S25 (Supporting Information) shows that the photocatalytic activity is still maintained at a high level even after five cycles of testing. The subsequent investigation focuses on the effect of the amount of photocatalyst on OWS. As demonstrated in Figure S26 (Supporting Information), when the catalyst dosage is 25 mg, the OWS activity is comparatively minimal. When the catalyst dosage is in the range of 25–100 mg, the OWS activity increases accordingly. Further increasing the dosage of the catalyst does not significantly improve the OWS activity. In principle, the presence of additional catalysts should result in an increased number of active sites, thereby enhancing OWS activity. However, it should be noted that, given the fixed volume of water, the introduction of additional catalysts will result in an increase in the concentration of catalysts within the system. The increased catalyst concentration may result in a light‐shielding effect, thereby affecting OWS activity. It is demonstrated that MCN‐4, with an intramolecular heterojunction, exhibits a superior photocatalytic hydrogen production half‐reaction activity under visible light irradiation (Figure 5d). This enhancement is attributed to the combination of the melon's visible light absorption and PTI's efficient electron transport properties.

**Figure 6:**
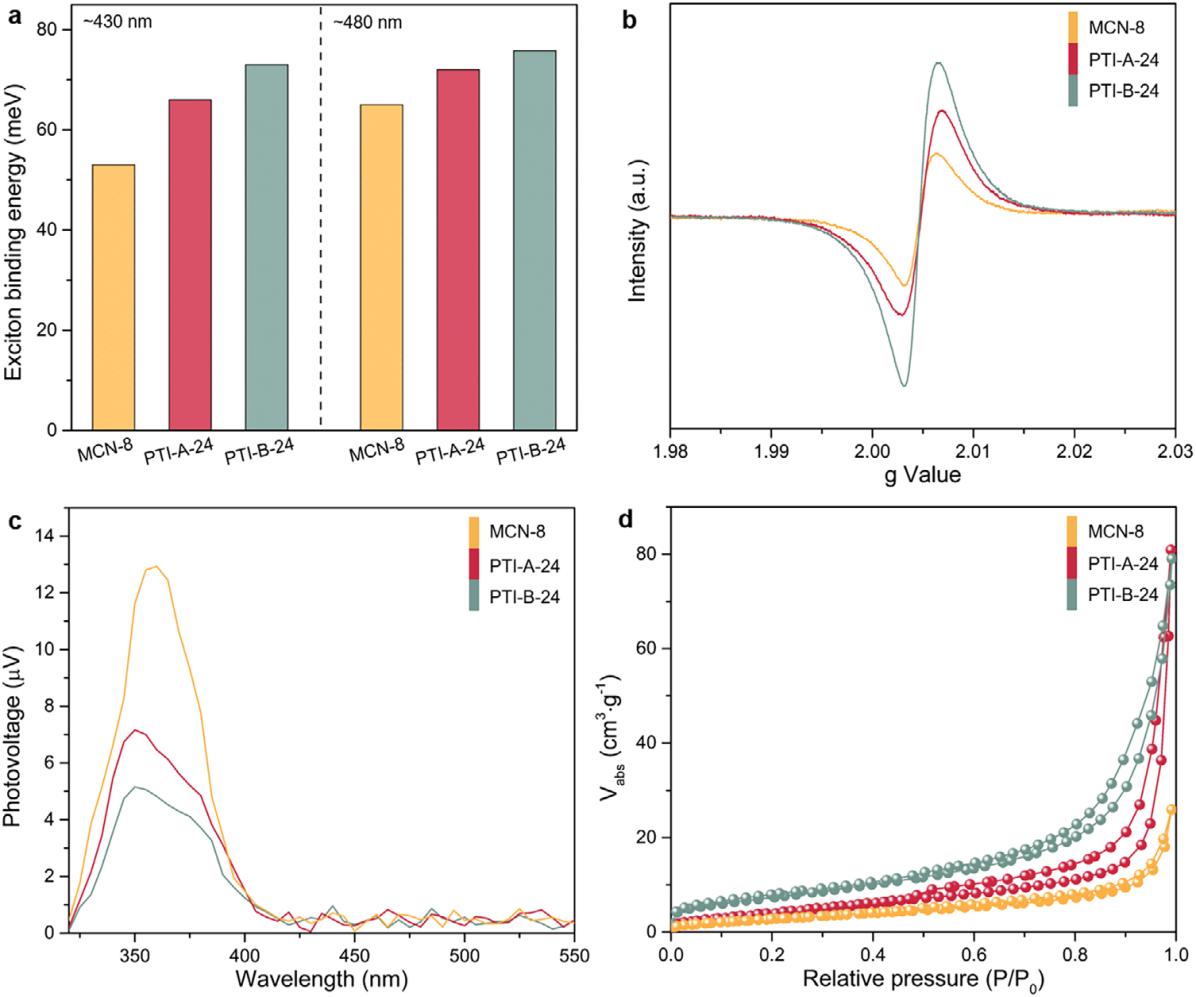
a) The fitted exciton binding energy of the samples. b) The room‐temperature EPR spectra of the samples. c) The SPV spectra of the samples. d) The N_2_ adsorption–desorption isotherms of the samples.

A series of characterization experiments was conducted to verify the effects of crystallinity and other factors on the properties of PTI‐based materials (MCN‐8, PTI‐A‐24, PTI‐B‐24). The temperature‐dependent PL spectra were then utilized to estimate the samples' exciton binding energy (*E_b_*).^[^ ^48^ ^]^ As shown in Figure S27 (Supporting Information), the PL spectra of the three samples exhibit two peaks near 430 and 480 nm, suggesting the presence of two pathways for the radiative recombination of electrons from the excited state (S_1_) to the ground state (S_0_). The phenomenon of PTI displaying multiple fluorescence emission peaks has also been documented in other reports.^[^ ^42^, ^49^ ^]^ To ensure the rigor of the data, the PL intensities at two wavelengths are fitted to obtain the corresponding *E_b_* values. Compared with PTI‐B‐24 (dicyandiamide as a precursor), PTI‐A‐24 and MCN‐8 (melon as a precursor) have smaller *E_b_* values ​​fitted at two wavelengths of fluorescence intensity (**Figure** **6**a). A smaller *E_b_* indicates that the exciton pairs are more readily separated into free electrons and holes, which is advantageous for participating in the subsequent OWS reaction.

Electron paramagnetic resonance (EPR) spectra were conducted to examine the local structure defects. As shown in Figure 6b, all samples show an obvious EPR response at a g‐value of 2.006, which is the typical signal of N vacancies.^[^ ^50^ ^]^ Among them, MCN‐8 exhibits the lowest EPR signal intensity, suggesting that the optimized synthesis process is capable of effectively mitigating structural defects. A reduction in structural defects, coupled with an extended conjugated system, has been demonstrated to improve charge separation and migration.^[^ ^27^, ^51^ ^]^ Therefore, steady‐state surface photovoltage (SPV) spectra are performed to investigate the separation/transfer efficiency of the photogenerated charges. As demonstrated in Figure 6c, the three samples show obvious positive SPV signals near 350 nm, which are attributed to the photogenerated holes that migrated to the catalyst surface. The SPV signal of MCN‐8 is stronger than that of PTI‐A‐24 and PTI‐B‐24, indicating that the concentration of holes accumulated on the surface is higher. Despite the reduction in specific surface area resulting from the large crystal size (Figure 6d; Table S5, Supporting Information), the high degree of crystallinity and the paucity of structural defects endow MCN‐8 with excellent charge separation and transport properties, thereby enhancing the OWS activity.

## 3 Conclusion

In summary, the synthetic protocol for PTI is rationally optimized through the utilization of melon‐type carbon nitride as a structural precursor, coupled with the elimination of pre‐calcination steps. This synthetic strategy endows MCN‐8 with a suite of advantageous structural attributes, including high crystallinity, large crystalline dimensions, minimized structural defects, and extended *π*‐conjugated frameworks, synergistically facilitating the separation and migration of photogenerated charge carriers. Significantly, MCN‐8 demonstrates superior photocatalytic OWS performance compared to conventionally prepared PTI counterparts, achieving a remarkable AQY of 13.1% under 365 nm monochromatic irradiation. This work establishes a scalable pathway for engineering carbon nitride‐based photocatalysts with atomic‐level precision, offering transformative potential for solar‐driven hydrogen production technologies.

## Conflict of Interest

The authors declare no conflict of interest.

## Supporting information



Supporting Information

## Data Availability

The data that support the findings of this study are available in the supplementary material of this article.
