# Supplementary material for: Large‐Sized Poly (Triazine Imide) Crystals with Minimized Defects for High‐Efficiency Overall Water Splitting
Source: Adv Sci (Weinh). 2025 Jul 29;12(40):e10084. doi: 10.1002/advs.202510084 (PMC12561331; doi:10.1002/advs.202510084)
Supplement: Supplementary file 1 — Supporting Information [file ADVS-12-e10084-s001.docx]

Large-Sized Poly (Triazine Imide) Crystals with Minimized Defects for High-Efficiency Overall Water Splitting

*Chong Wang ^a, b^*^#^, Na Shi ^a#^, Yulin Zhou ^a#^, Yichun Lu ^c^, Jingru Zhuang ^b^, Hongwu Liao ^a^,* *Chengning Ye* *^a^, Hanhui Lei ^d^, Xiangfeng Lin ^a^*, Jiaxian Zheng ^a^*, Terence Xiaoteng Liu ^d^*, Zhanhui Yuan ^a^**

1. College of Materials Engineering, Fujian Agriculture and Forestry University, Fuzhou 350002, China. E-mail: [zhanhuiyuan@fafu.edu.cn](mailto:zhanhuiyuan@fafu.edu.cn); xiangfenglin@fafu.edu.cn; [zjxcms@fafu.edu.cn](mailto:zjxcms@fafu.edu.cn)
2. Department of Chemistry, The Chinese University of Hong Kong, Shatin, Hong Kong SAR 999077, China. E-mail: [chongwang@cuhk.edu.hk](mailto:chongwang@cuhk.edu.hk)
3. State Key Laboratory of Marine Pollution, City University of Hong Kong, Kowloon Tong, Hong Kong SAR 999077, China.
4. Department of Mechanical and Construction Engineering, Northumbria University, Newcastle-upon-Tyne NE1 8ST, U.K. E-mail: [terence.liu@northumbria.ac.uk](mailto:terence.liu@northumbria.ac.uk)

*^#^* Those authors were contributed to the work equally.

**S1 Experimental section**

**S1.1 Material**

Dicyandiamide (C_2_H_4_N_4_, 99%) was purchased from Acros. Lithium chloride (LiCl, 99%) and potassium chloride (KCl, 99%) were purchased from Alfa Aesar Chemicals Co. Ltd. All the materials were used without further purification.

**S1.2 Catalyst synthesis**

**S1.2.1 BCN**

8 g of dicyandiamide was heated to 550 ℃ in an air atmosphere (muffle furnace) at a heating rate of 6 ℃·min^-1^ and kept for 4 h to obtain the faint yellow solid.

**S1.2.2 MCN samples**

1 g of the preheated BCN was ground with KCl (6.6 g) and LiCl (5.4 g) in the glove box. The mixture was transferred to an open glass tube, and the glass tube was sealed and heated to 550 °C for different hours at a rate of 6 ℃·min^-1^ in a tube furnace. After cooling to room temperature, the samples were washed with boiling deionized water several times and dried at 60 ℃ under vacuum. These samples were denoted as MCN-T (where T refers to the calcination time).

**Note:** For safety instructions, an explosion may happen if too much pressure is generated during the heating process due to the evolution of large amounts of ammonia and other gases.

**S1.2.3 PTI-A**

1 g of the preheated BCN was ground with KCl (6.6 g) and LiCl (5.4 g) in the glove box. Then, the mixture was transferred to an open glass tube and heated to 400 ℃. After cooling to room temperature, the glass tube was sealed and heated to 550 ℃ for 8 or 24 hours at 6 ℃·min^-1^ in a tube furnace. After cooling to room temperature, the sample was washed with boiling deionized water several times and dried at 60 ℃ under vacuum.

**Note:** For safety instructions, an explosion may happen if too much pressure is generated during the heating process due to the evolution of large amounts of ammonia and other gases.

**S1.2.4 PTI-B**

1 g dicyandiamide was ground with KCl (6.6 g) and LiCl (5.4 g) in the glove box. Then, the mixture was transferred to an open glass tube and heated to 400 ℃. After cooling to room temperature, the glass tube was sealed and heated to 550 ℃ for 8 or 24 hours at 6 ℃·min^-1^ in a tube furnace. After cooling to room temperature, the sample was washed with boiling deionized water several times and dried at 60 ℃ under vacuum.

**Note:** For safety instructions, an explosion may happen if too much pressure is generated during the heating process due to the evolution of large amounts of ammonia and other gases.

**S1.3 Characterizations**

Powder X-ray diffraction (PXRD) measurements were performed on a powder X-ray diffractometer at 40 kV and 15 mA using Cu Kα radiation (Miniflex, Rigaku). Fourier transform infrared (FT-IR) spectra were acquired on a Nicolet IS-50 instrument. Elemental analysis (EA) results were collected from a Vario EL Cube. X-ray photoelectron spectroscopy (XPS) was performed on an ESCA Lab 250 X-ray photoelectron spectrometer (Thermo-VG Scientific, America). All binding energies were calibrated by the internal standard C 1s peak (284.80 eV) that arises from surface adventitious carbon. The solid-state ^13^C NMR experiments were performed on Bruker Advance III 500 spectrometers. The morphology of the sample was investigated by field emission scanning electron microscopy (SEM) (JSM-6700F). Transmission electron microscopy (TEM) was obtained by a Zeiss 912 microscope. The samples' UV-Vis diffuse reflectance spectra (DRS) were conducted on a Varian Cary 500 Scan UV-Vis spectrophotometer with barium sulfate as the reference. Photoluminescence (PL) spectra were collected on an Edinburgh FI/FSTCSPC 980 spectrophotometer. Metal contents were measured by inductively coupled plasma-optical emission spectrometer (ICP-OES) (PerkinElmer, Avio 200).

**S1.4 Electrochemical Measurements**

The photoelectrochemical measurements, including photocurrent response, Mott-Schottky plots, and electrochemical impedance spectra (EIS), were conducted on an electrochemical workstation (CHI 670E, Chenhua, Shanghai) with a standard three-electrode cell using a Pt plate as a counter electrode, a KCl-saturated (3 mol·L^-1^) Ag/AgCl electrode as reference electrode and the sample deposited F-doped SnO_2_-coated (FTO) glass as working electrode, and 0.5 mol·L^-1^ Na_2_SO_4_ (pH = 7) aqueous solution as the electrolyte. The working electrodes were synthesized as follows: 10 mg of the sample was dispersed in 0.8 mL of dimethylformamide (DMF) by sonication to give a slurry mixture. 20 μL of the slurry was spread onto pretreated FTO glass. The catalyst-coated FTO was naturally dried overnight and subjected to a 2h calcination process at 150 ℃ in an Ar atmosphere to increase the adhesion force between them.

**S1.5 Photocatalytic hydrogen production**

The reactions were carried out in a Pyrex top-irradiation reaction vessel connected to a glass-closed gas system (Labsolar-6A, Beijing Prefectlight Technology). The hydrogen production was performed by dispersing 50 mg of photocatalyst powder in pure water (100 mL) containing 10% vol (10 mL) triethanolamine (TEOA) as the sacrificial agent. Then, 3 wt.% Pt was loaded on the catalyst's surface by in situ photo-deposition method using H_2_PtCl_6_ as the co-catalyst. The reaction solution was evacuated several times to remove the air before 300 W Xe lamp irradiation (15 A, λ > 420 nm). The wavelength of the incident light was controlled by using an appropriate long-pass cut-off filter. The reaction solution temperature was maintained at 15 ℃ using a cooling water flow during the reaction. The produced gases were analyzed with a gas chromatograph (Shimadzu 8A) equipped with a thermal conductivity detector (TCD) with argon as the carrier gas.

**S1.6 Photocatalytic overall water splitting**

The photocatalyst (100 mg) was well-dispersed in 100 mL of pure water. Pt and CoO_x_ were deposited on the surface of PTI samples as hydrogen and oxygen evolution cocatalysts, respectively. Cocatalyst deposition was conducted as follows: a certain amount of CoCl_2_ (3 wt% based on Co atoms) was first added into the solution after being irradiated with light for 1 h to ensure all cobalt atoms were in situ deposited on the surface of the catalyst. Then, H_2_PtCl_6_ (1 wt% based on Pt atoms) was added to the solution and irradiated for another 1 h. After loading the cocatalysts, the reaction solution was irradiated with the full spectrum of a 300 W Xe lamp (15 A, λ > 300 nm). The spectral range of the Xe lamp is shown in the following Figure. The temperature of the reaction solution was maintained at 15 ℃ by a cooling water system. The produced gases were quantified using a gas chromatograph (Shimadzu GC-8A, TCD, argon carrier gas).


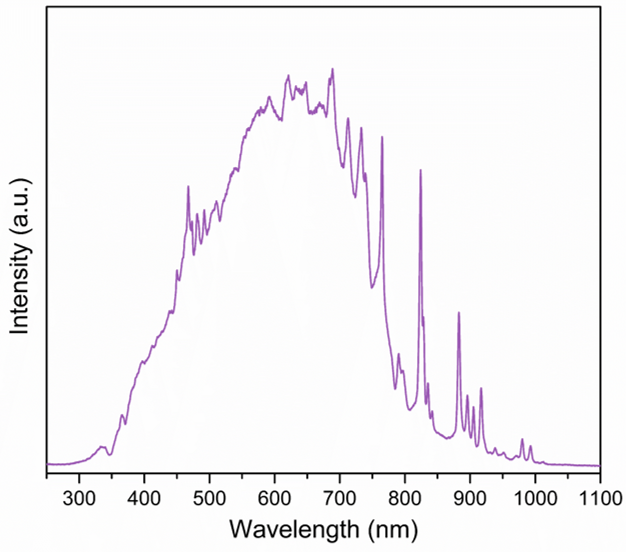


The spectral range of the Xe lamp.

**S1.7** **Apparent quantum efficiency (AQY) tests**

The reactions were carried out in a Pyrex top-irradiation reaction vessel connected to a glass closed gas system. 100 mg of the sample loaded with the co-catalyst was dispersed in 100 mL of water. The reaction solution was evacuated several times to completely remove the air. The AQY for overall water splitting was carried out under the irradiation of various monochromatic lights using different wavelength LED lamps (365, 380, and 405 ±10 nm) with specific band-pass filters for 60 min. The average light intensity is measured by the optical power meter. The following equation calculates the AQY:

$$\text{AQY}\text{ }\text{=}\text{ }\frac{\text{N}_{\text{e}}}{\text{N}_{\text{p}}}\text{ }\text{×}\text{ }\text{100 \%}\text{ }\text{=}\text{ }\frac{\text{2}\text{ }\text{×}\text{ }\text{M}\text{ }\text{×}\text{ }\text{N}_{\text{A}}}{\frac{\text{E}_{\text{total}}}{\text{E}_{\text{photon}}}}\text{ }\text{×}\text{ }\text{100 \%}\text{ }\text{=}\text{ }\frac{\text{2}\text{ }\text{×}\text{ }\text{M}\text{ }\text{×}{\text{ }\text{N}}_{\text{A}}}{\frac{\text{S}\text{ }\text{×}\text{ }\text{P}\text{ }\text{×}\text{ }\text{t}}{\text{h}\text{ }\text{×}\text{ }\frac{\text{c}}{\text{λ}}}}\text{ }\text{×}\text{ }\text{100 \%}\text{ }\text{=}\text{ }\frac{\text{2}\text{ }\text{×}\text{ }\text{M}\text{ }\text{×}{\text{ }\text{N}}_{\text{A}}\text{ }\text{×}\text{ }\text{h}\text{ }\text{×}\text{ }\text{c}}{\text{S}\text{ }\text{×}\text{ }\text{P}\text{ }\text{×}\text{ }\text{t}\text{ }\text{×}\text{ }\text{λ}}\text{ }\text{×}\text{ }\text{100 \%}$$

Where M is the number of H_2_ molecules (mol), N_A_ is the Avogadro constant (6.022×10^23^ mol^-1^), *h* is the Planck constant (6.626×10^-34^ J·s), c is the speed of light (3×10^8^ m·s^-1^), S is the irradiation area (9 cm^2^), P is the intensity of irradiation light (W·cm^-2^), t is the photoreaction time (3600 s), λ is the wavelength of the monochromatic light (m). The light intensities of 365, 380, and 405 nm LED lamps are 5.6, 6.8, and 4.7 mW·cm^-2^, respectively. Three parallel experiments were carried out to verify the reproducibility of some experiments. Error bars in the figures correspond to the standard deviation (*n* = 3).

**S2 Supernumerary figures and tables**


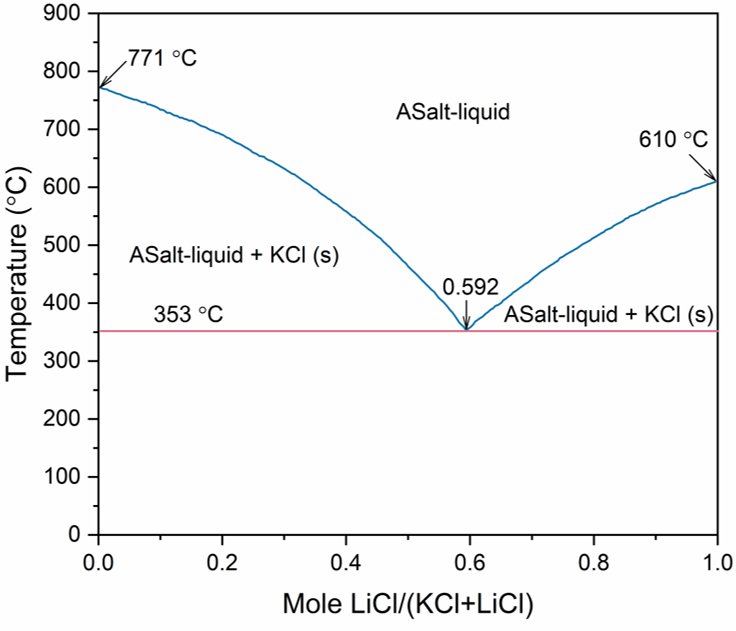


**Figure S1.** The phase diagram of the eutectic point (Data from FTsalt ⋅ FACT salt databases).

**
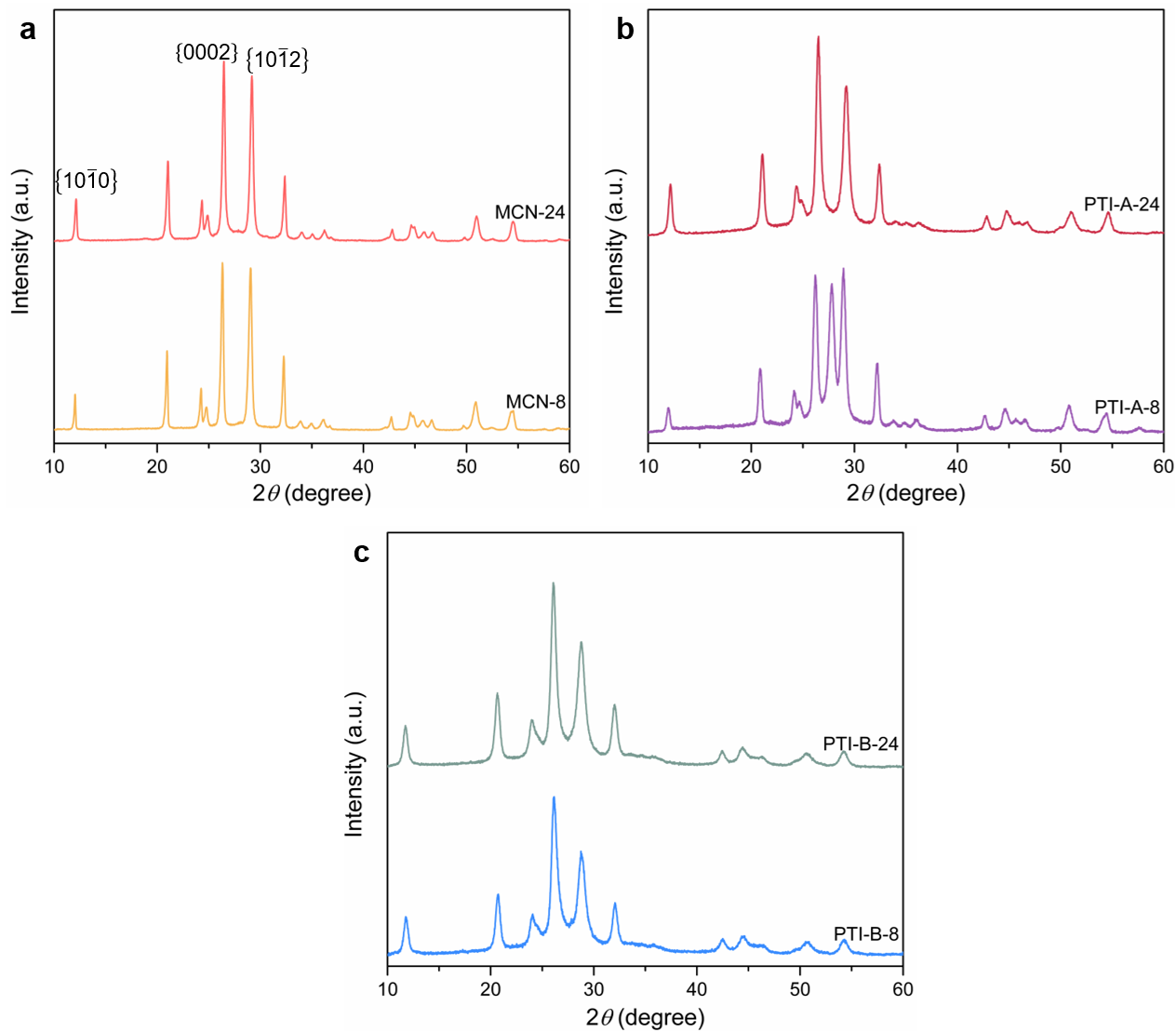
**

**Figure S2.** The XRD patterns of (a) MCN samples, (b) PTI-A samples, and (c) PTI-B samples.

**
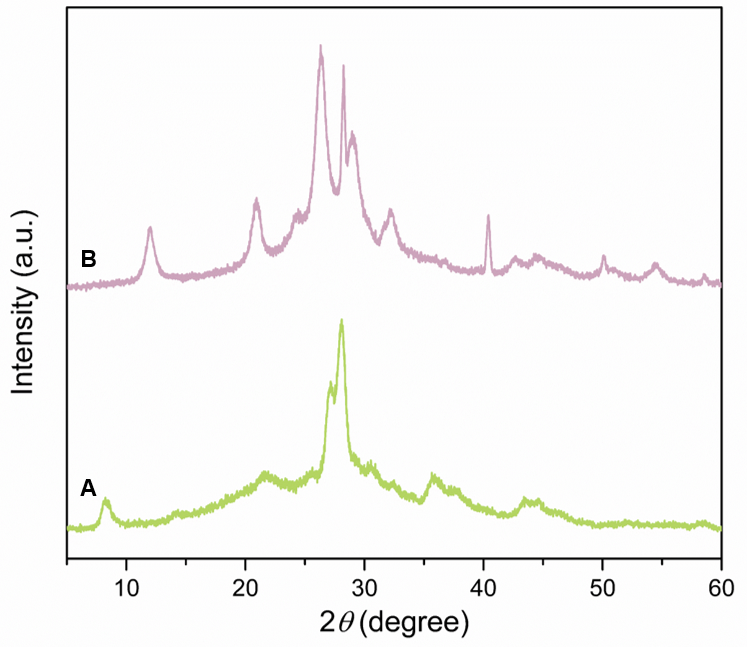
**

**Figure S3.** The XRD patterns of the samples. (**A**) 1 g of melon was ground with KCl (6.6 g) and LiCl (5.4 g) in the glove box. Then, the mixture was transferred to a crucible and heated to 550 ℃ for 8 or 24 hours at 6 ℃·min^-1^ in a tube furnace. After cooling to room temperature, the sample was washed with boiling deionized water several times and dried at 60 ℃ under vacuum. (**B**) 1 g of dicyandiamide was ground with KCl (6.6 g) and LiCl (5.4 g) in the glove box. Then, the mixture was transferred to a crucible and heated to 550 ℃ for 8 or 24 hours at 6 ℃·min^-1^ in a tube furnace. After cooling to room temperature, the sample was washed with boiling deionized water several times and dried at 60 ℃ under vacuum.

**
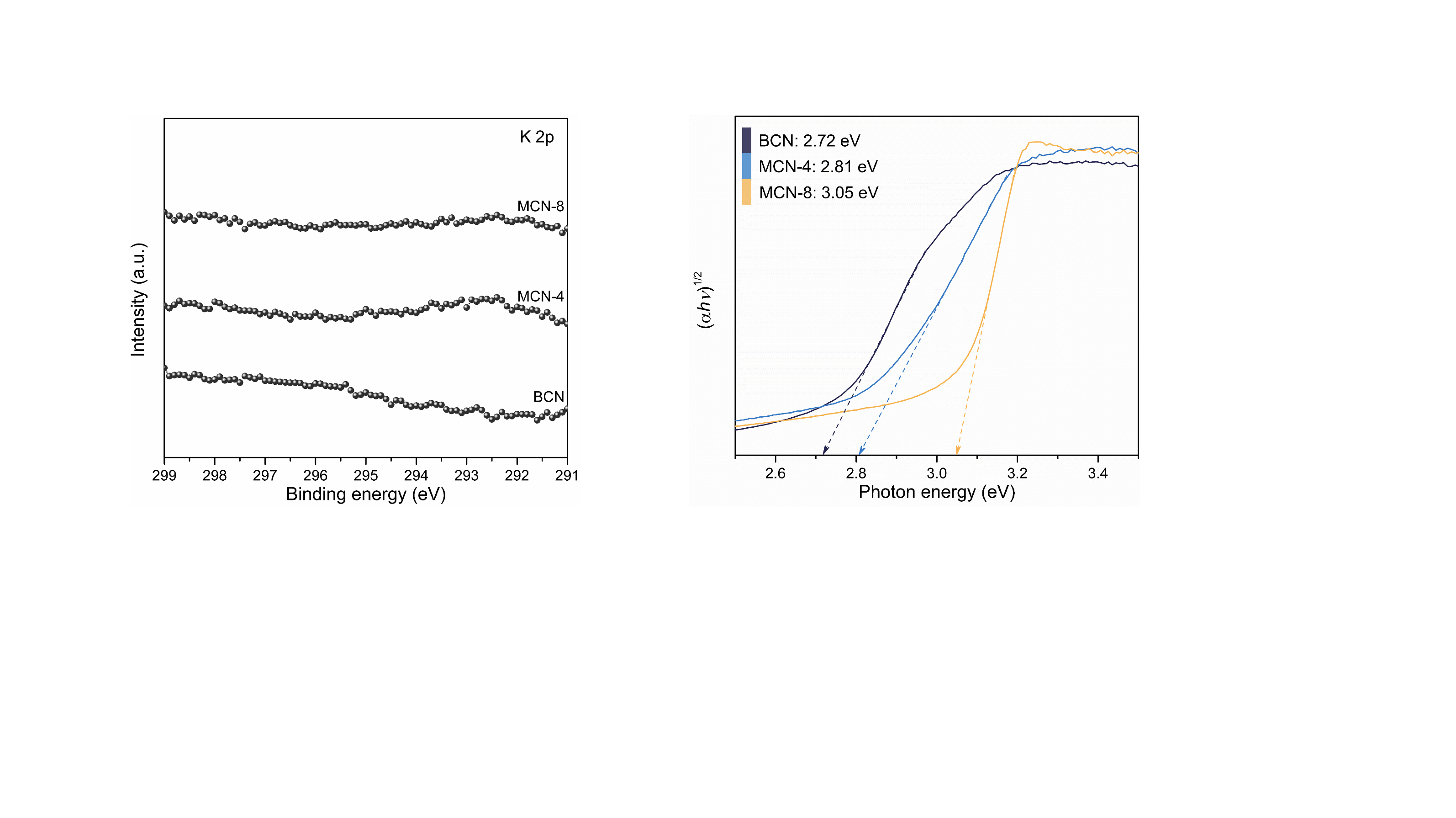
**

**Figure S4.** High-resolution XPS spectra of K 2p for the samples.

**
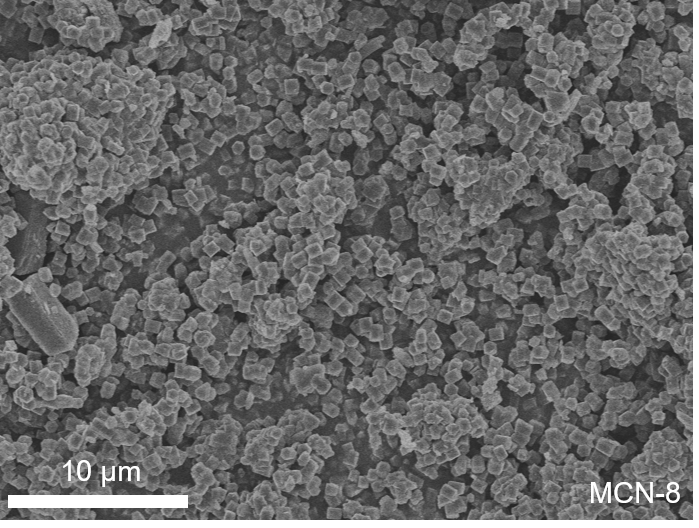
**

**Figure S5.** The SEM image of MCN-8.


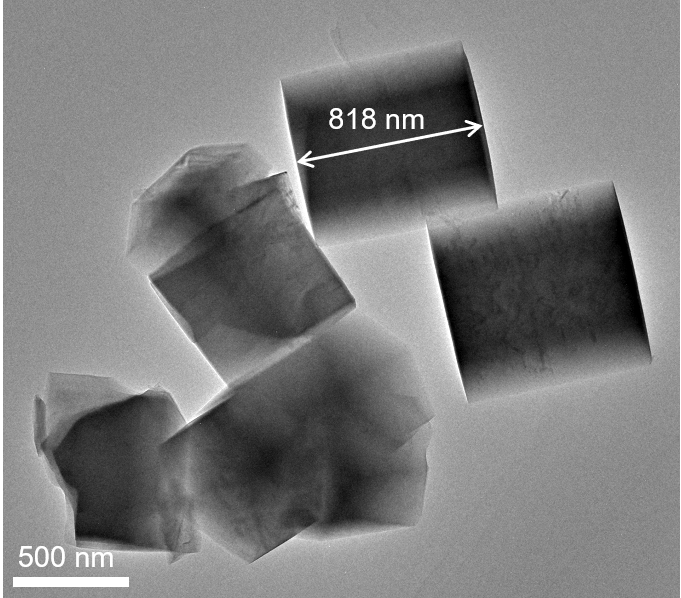


**Figure S6.** The TEM image of MCN-8.


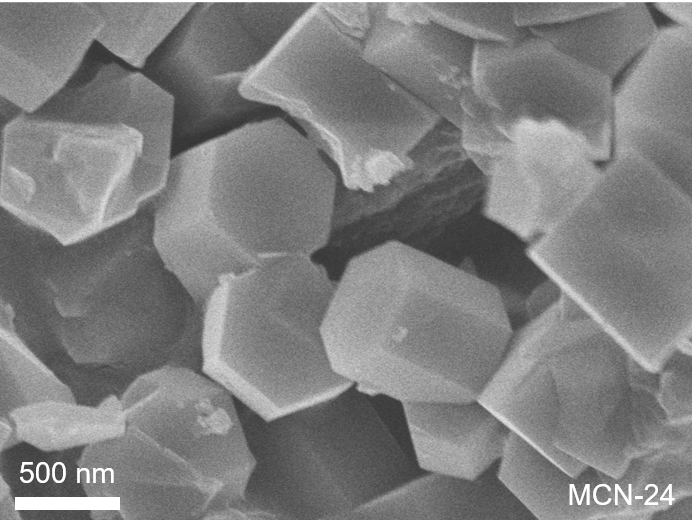


**Figure S7.** The SEM image of MCN-24.

**
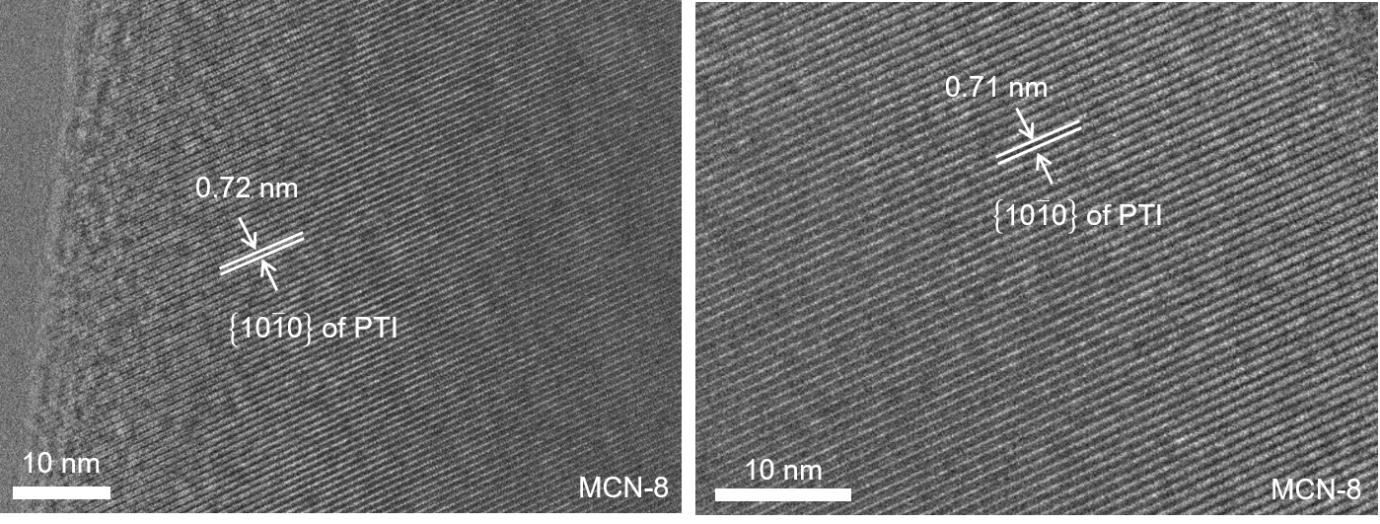
**

**Figure S8.** The HR-TEM images of MCN-8.

**
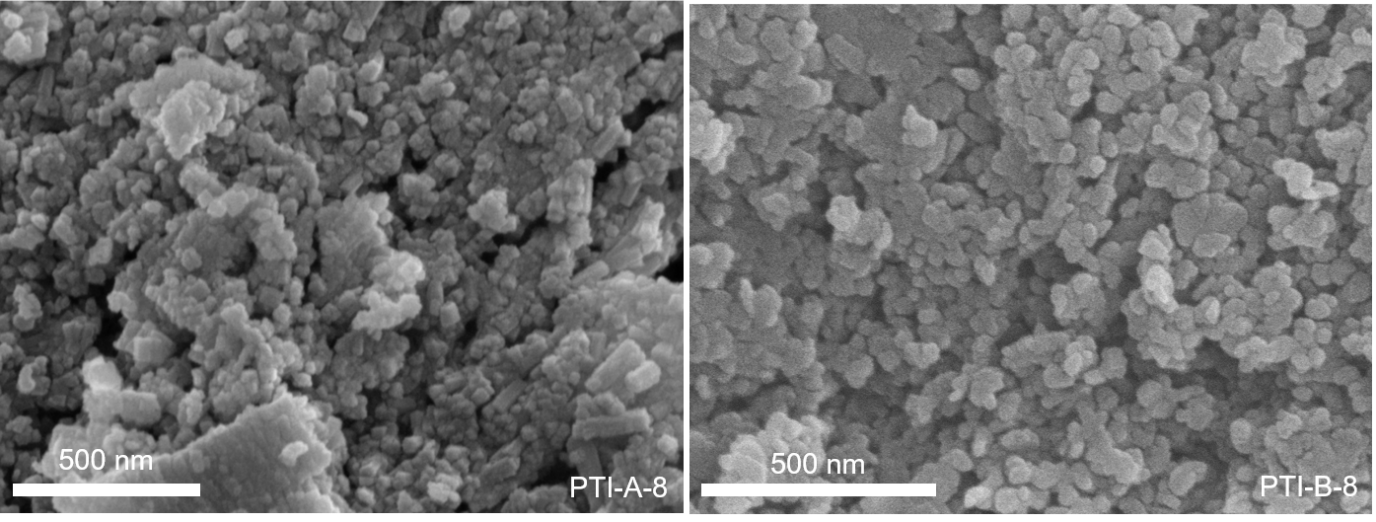
**

**Figure S9.** The SEM images of PTI-A-8 and PTI-B-8.

**
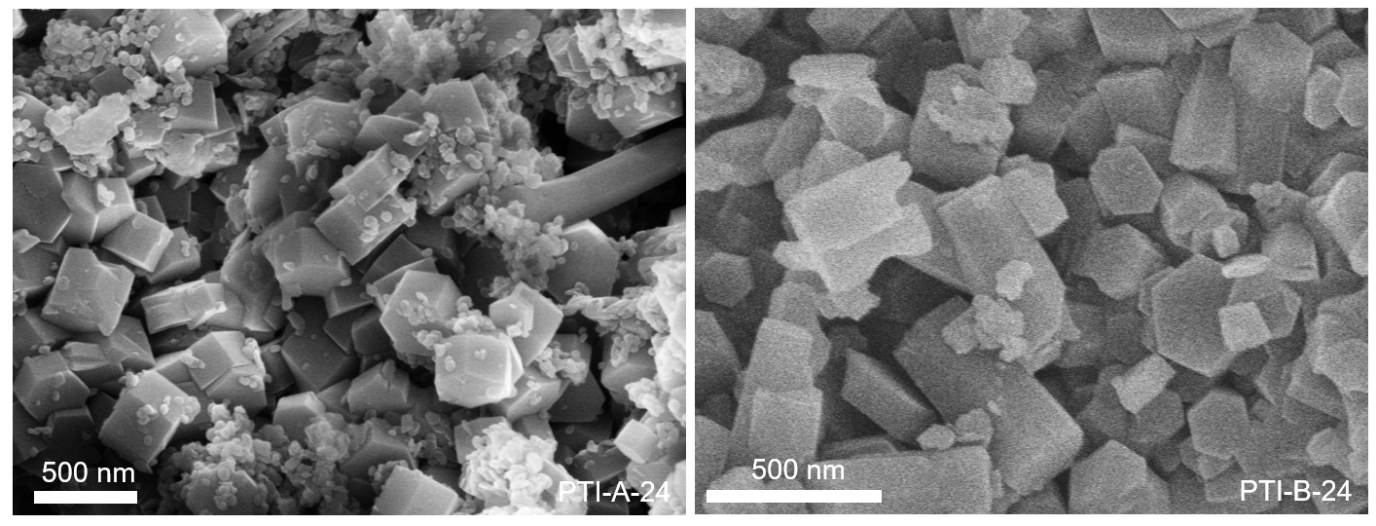
**

**Figure S10.** The SEM images of PTI-A-24 and PTI-B-24.

**
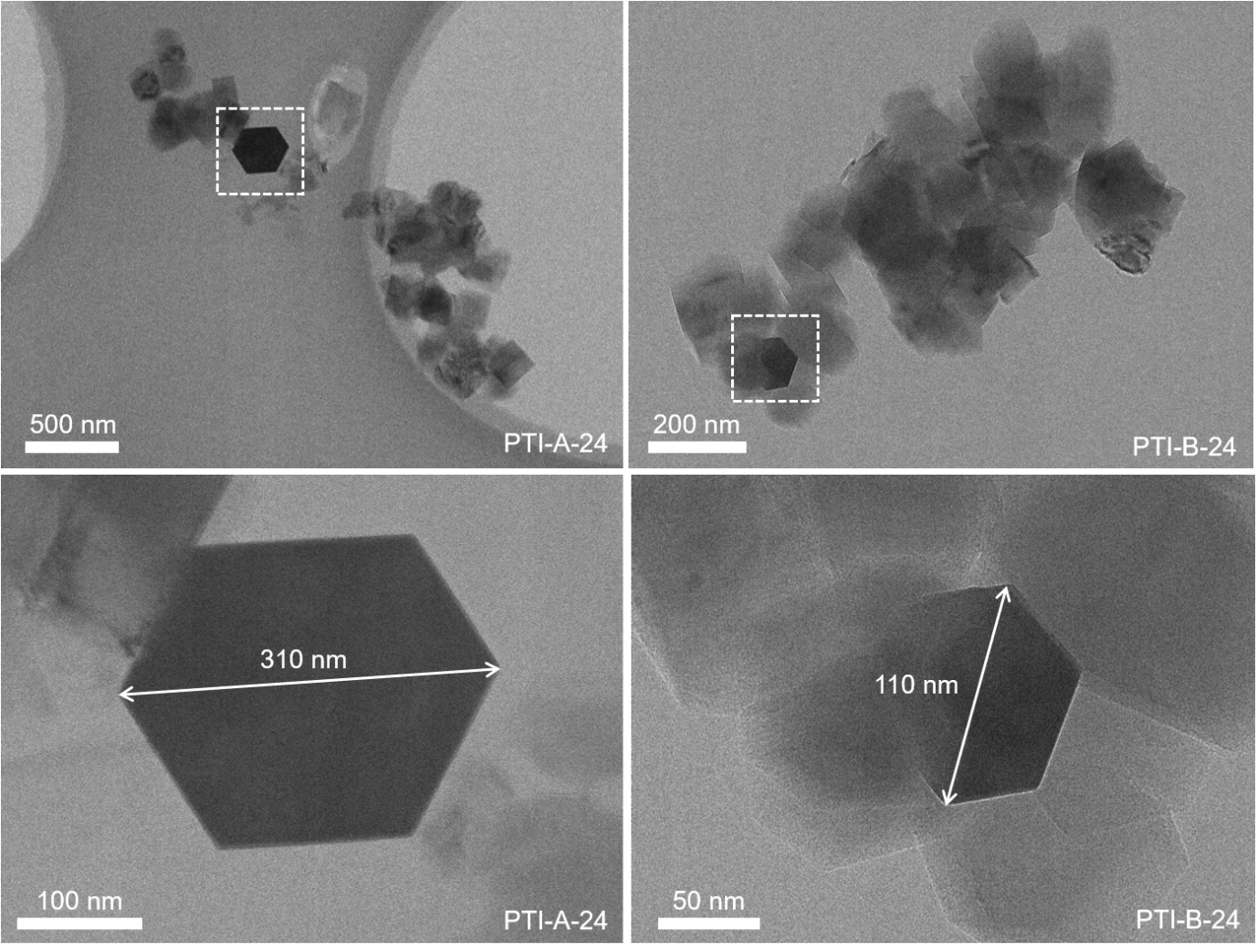
**

**Figure S11.** The TEM images of PTI-A-24 and PTI-B-24.


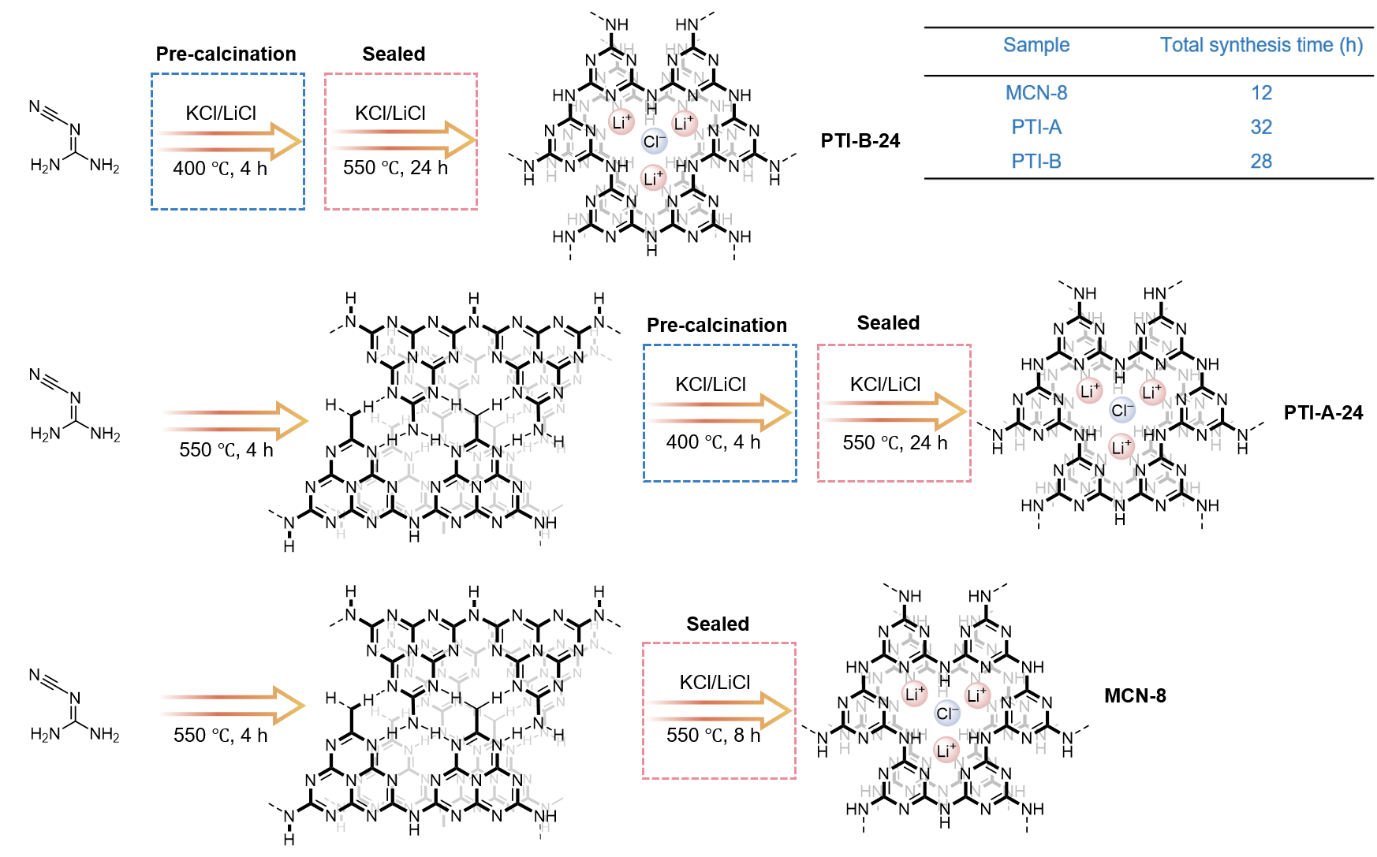


**Scheme S1.** The synthesis steps and calcination time of the samples.


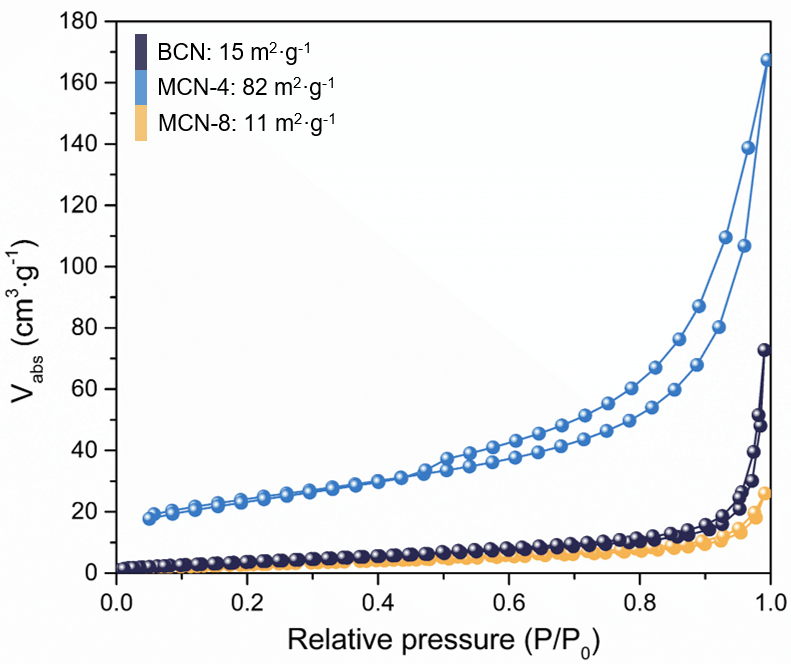


**Figure S12.** The N_2_ adsorption-desorption isotherms of the samples.

**
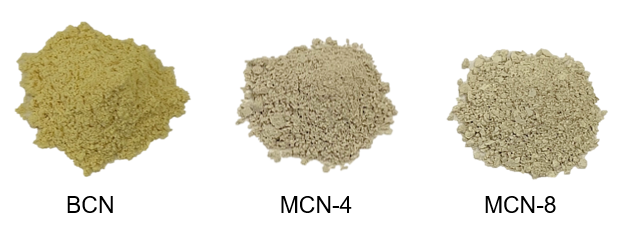
**

**Figure S13.** The photographs of the samples.


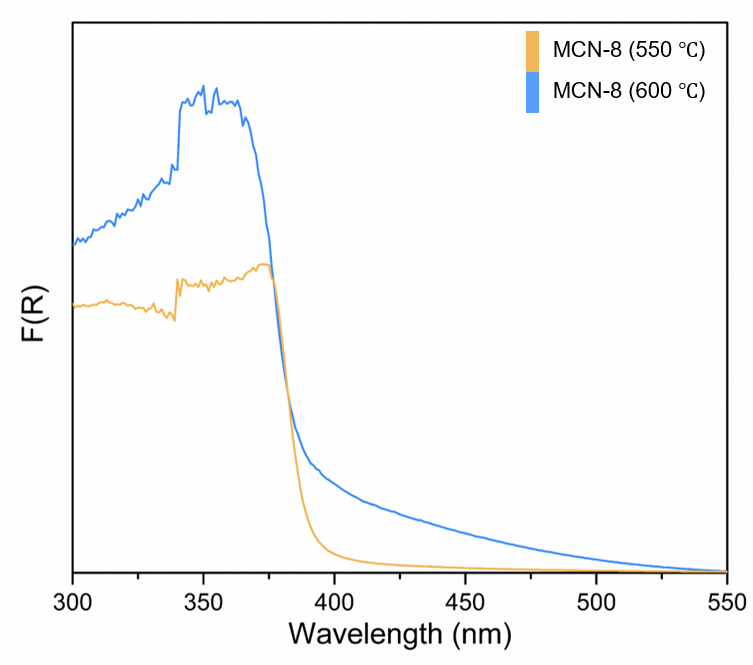


**Figure S14.** The UV-Vis DRS of the samples.


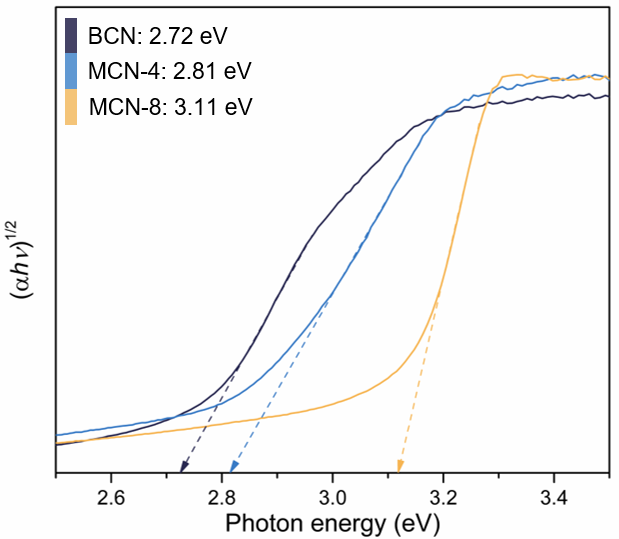


**Figure S15.** The band gap energies of the samples.


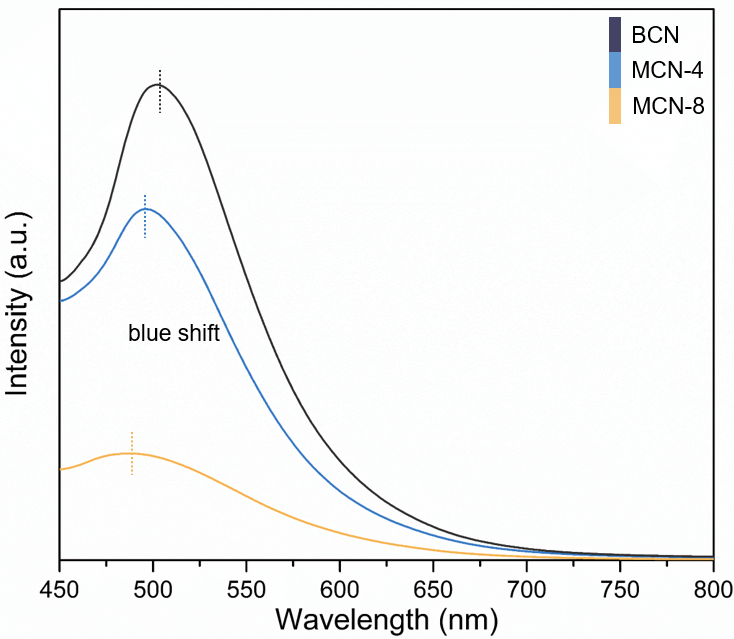


**Figure S16.** The PL spectra under 420 nm excitation.

**
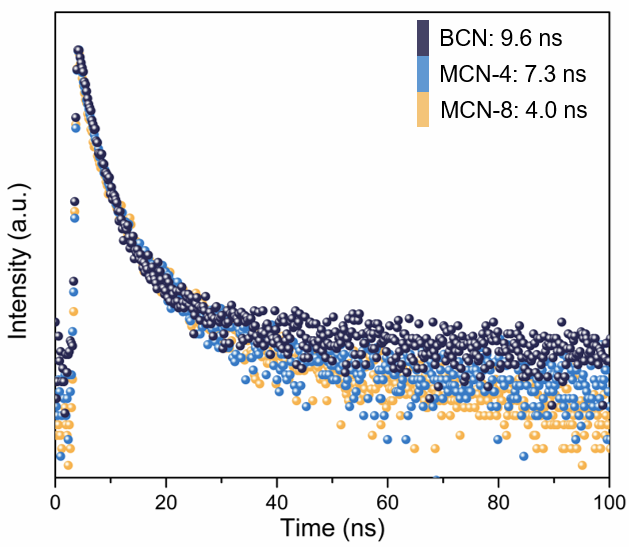
**

**Figure S17.** The PL lifetime of the samples.


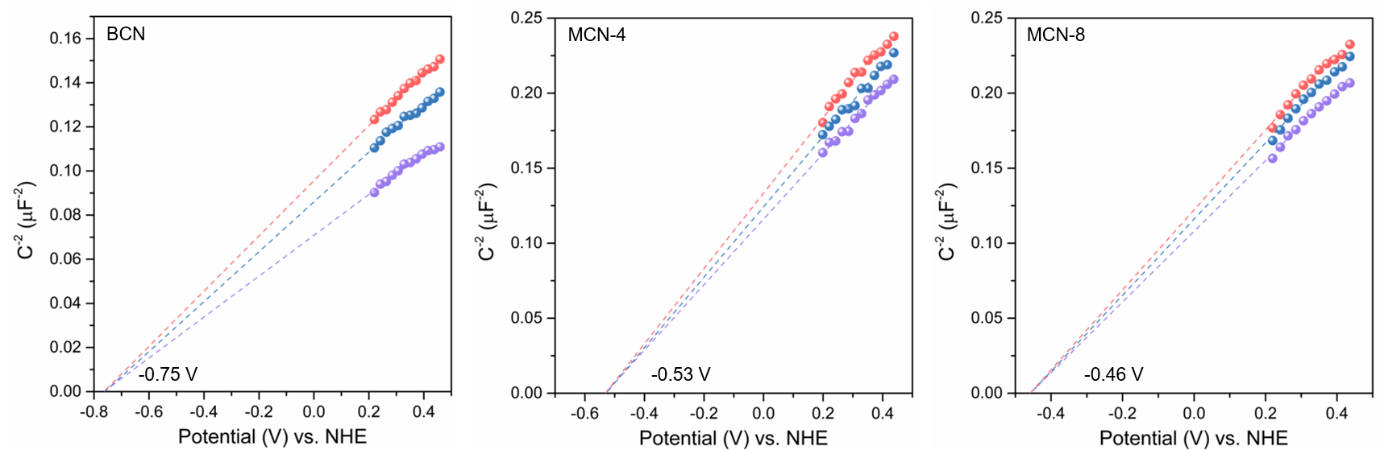


**Figure S18.** Mott-Schottky plots of the samples.

*E*_(vs. NHE)_ = *E*_(vs. Ag/AgCl)_ + *E*^º^_Ag/AgCl (vs. NHE)_ = *E*(_vs. Ag/AgCl_) + 0.197 V

$$\text{E}_{\text{C}}\text{ }\text{=}\text{ }\text{E}_{\text{f}\text{b }}-\text{ }\text{∆E}_{\text{H}\text{ }}- \frac{\text{k}\text{T}}{\text{e}}\left( \frac{\text{N}_{\text{D}}}{\text{n}_{\text{i}}} \right)$$

*E_C_* ≈ *E_fb_* – 0.1 V


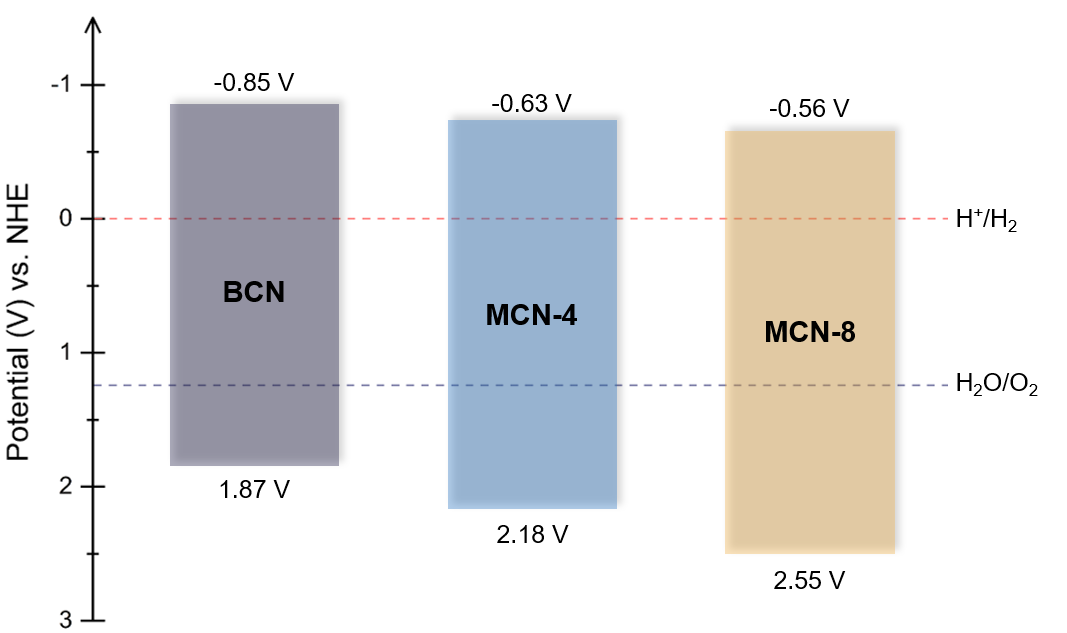


**Figure S19**. Schematic illustration of the band structures.


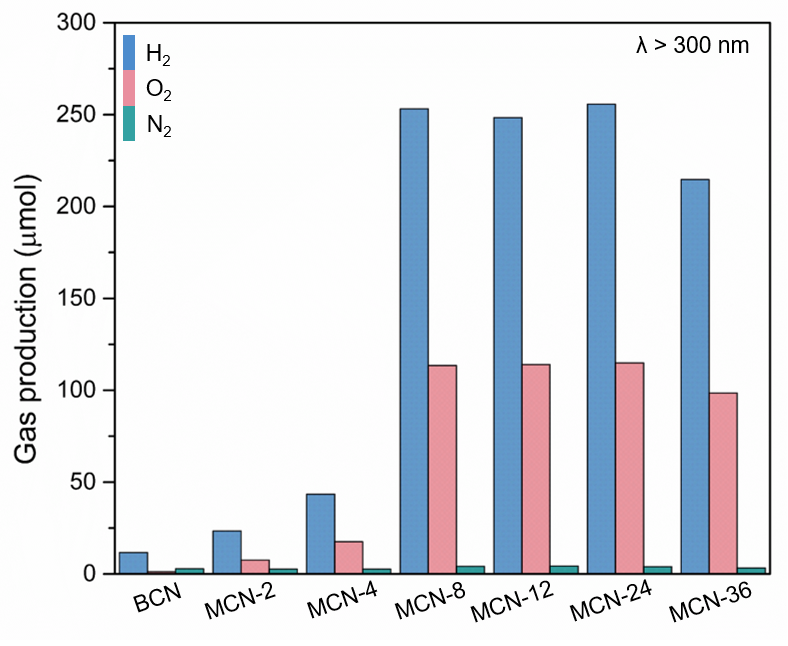


**Figure S20**. The photocatalytic OWS activities of the samples (300 W Xe lamp, λ > 300 nm, 1 h).


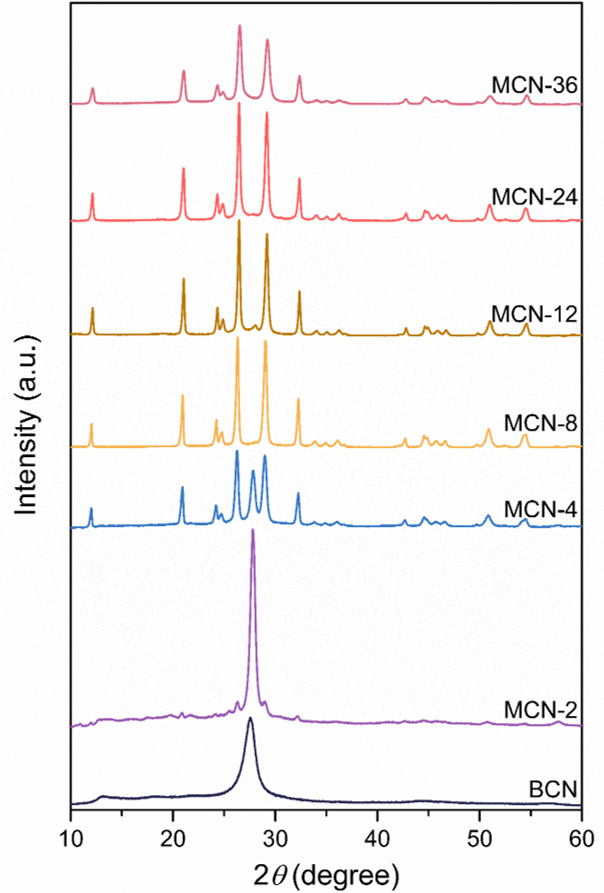


**Figure S21**. The XRD patterns of the samples.


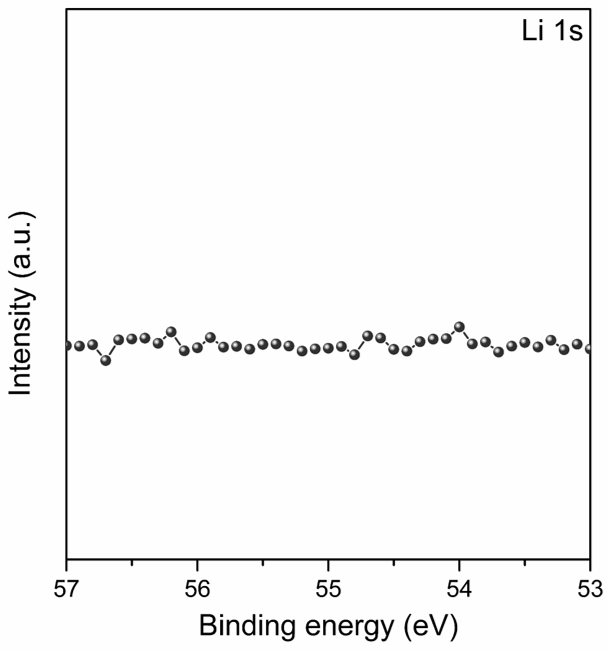


**Figure S22**. The high-resolution Li 1s XPS spectrum of HMCN-8 (MCN-8 washed by 0.1 M HCl).


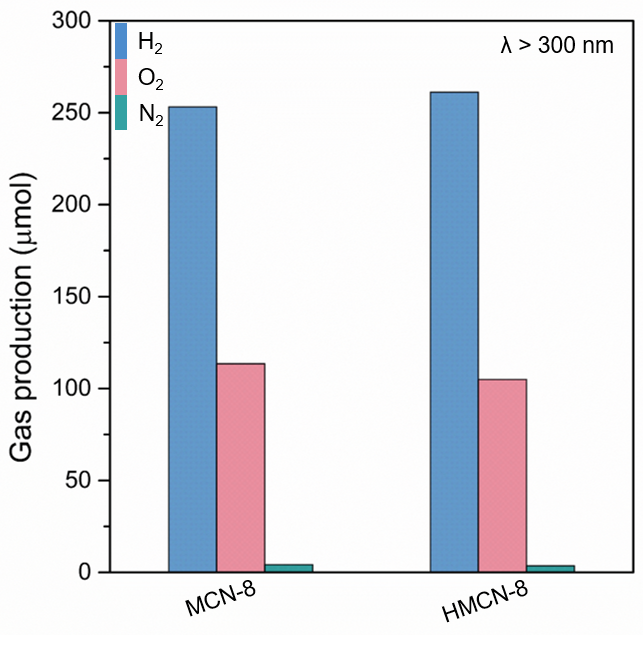


**Figure S23**. The photocatalytic OWS activities of the samples (300 W Xe lamp, λ > 300 nm, 1 h).


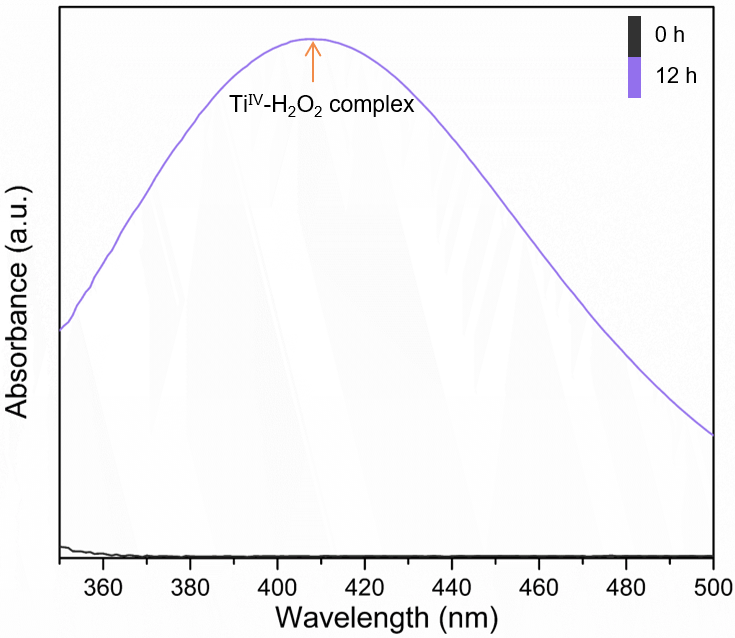


**Figure S24**. UV-vis absorption spectra of Ti(SO_4_)_2_ after reacting with the reaction solution.

**
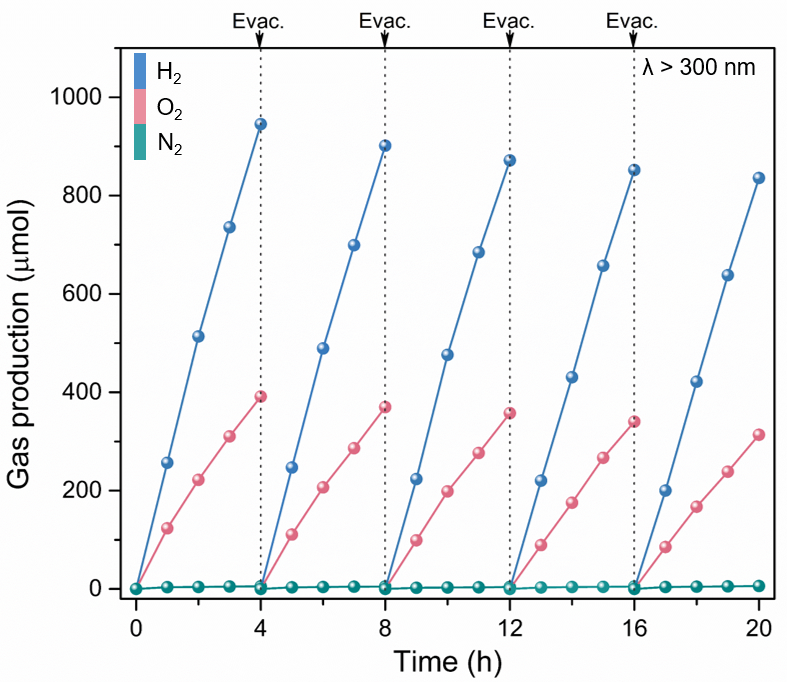
**

**Figure S25**. Time course of overall water splitting over MCN-8 (300 W Xe lamp, λ > 300 nm).


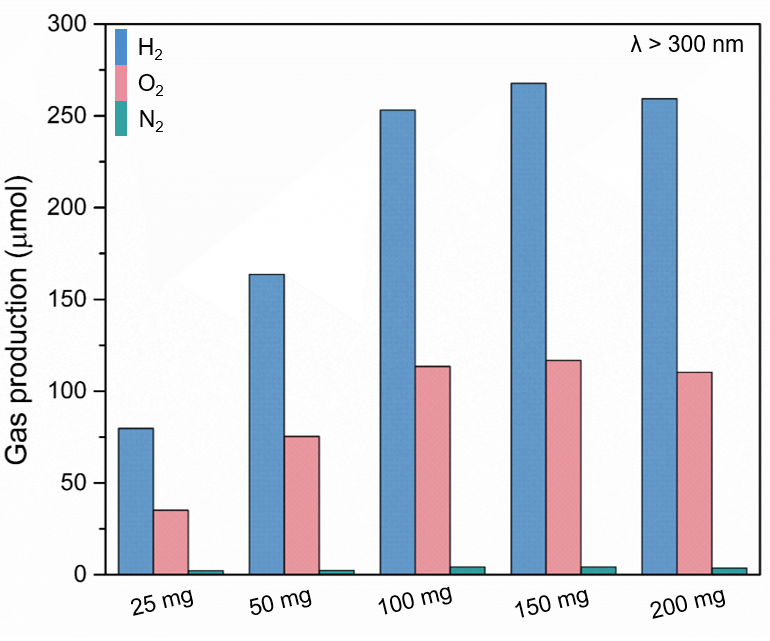


**Figure S26**. The catalyst amount-dependent overall water splitting over MCN-8 (300 W Xe lamp, λ > 300 nm, 1 h).


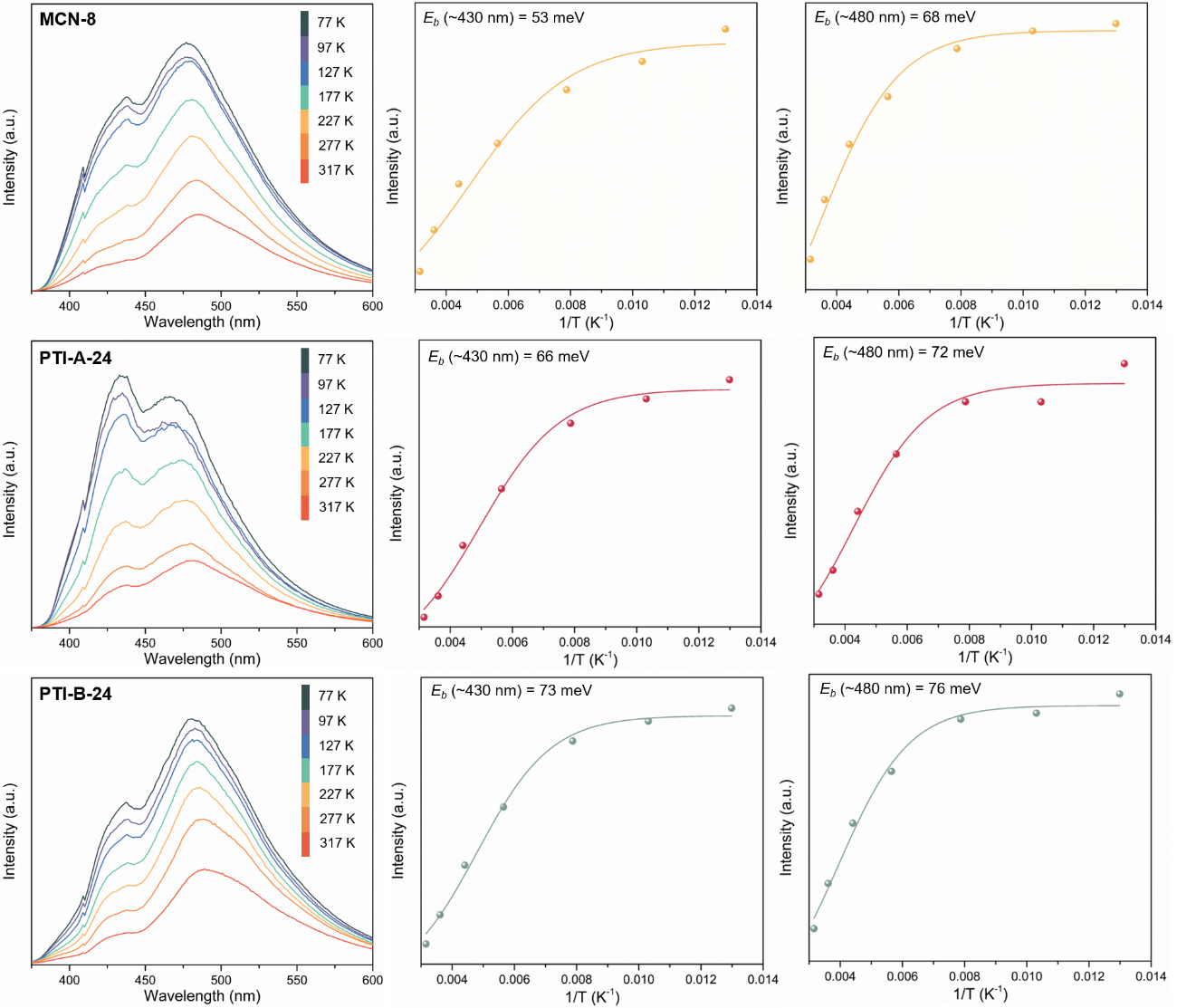


**Figure S27.** Temperature-dependent PL spectra and the integrated PL emission intensity of samples.

**Table S1.** Detailed information on XRD tests.

| **Sample** | **FWHM (**^o^**)^[a]^**  $\left( \text{10}\bar{\text{1}}\text{0} \right)$ | **FWHM (**^o^**)**  $\left( 0002 \right)$ | **FWHM (**^o^**)**  $\left( 10\bar{1}2 \right)$ |
| --- | --- | --- | --- |
| MCN-8 | 0.15 | 0.31 | 0.39 |
| PTI-A-24 | 0.38 | 0.63 | 0.83 |
| PTI-B-24 | 0.51 | 0.84 | 1.31 |
| B in Figure S2 | 0.94 | 1.35 | 1.55 |

[a] FWHM = full width at half maxima.

**Table S2.** Elemental analysis of the samples.

| **Sample** | **C (wt%)** | **N (wt%)** | **H (wt%)** | **C/N** |
| --- | --- | --- | --- | --- |
| BCN | 34.85 | 62.25 | 2.22 | 0.65 |
| MCN-4 | 33.87 | 60.07 | 2.07 | 0.66 |
| MCN-8 | 29.37 | 51.27 | 1.31 | 0.67 |

**Table S3.** Amounts of cocatalyst loaded on PTI obtained from ICP-OES.

| **Sample** | **Pt (wt%)** | **Co (wt%)** |
| --- | --- | --- |
| Pt/CoOx-MCN-8 | 0.934 | 1.646 |

**Table S4.** Representative one-step excitation overall water-splitting performance of PTI-based photocatalysts.

| **Sample** | **Precursor** | **Molten salt** | **Cocatalyst** | **AQY/AQE** | **Particle size** | **Ref.** |
| --- | --- | --- | --- | --- | --- | --- |
| PTI/H^+^Cl^-^ | Dicyandiamide | KCl/LiCl | Pt, CoO_x_ | 2.1% at 365 nm | ~50 nm | R1 |
| PTI-550 | Dicyandiamide | KCl/LiCl | Pt, CoO_x_ | 8.0% at 365 nm | ~183 nm | R2 |
| PTI-LiNa | Dicyandiamide | NaCl/LiCl | Pt, CoO_x_ | 12.0% at 365 nm | ~43 nm | R3 |
| PTI-LiNa | Dicyandiamide | NaCl/LiCl | CoO_x_, Rh, Cr_2_O_3_ | 20.2% at 365 nm | ~341 nm | R4 |
| KPTI | Melamine | KCl/LiCl/KSCN | Pt, CoO_x_ | 14.81% at 350 nm | ~61 nm | R5 |
| PTI-MC | Melamine, cyanuric acid | NaCl/LiCl | CoO_x_, Rh, Cr_2_O_3_ | 21.2% at 365 nm | ~104 nm | R6 |
| PTI-LiNaK | Dicyandiamide | KCl/LiCl/NaCl | CoO_x_, Rh, Cr_2_O_3_ | 25% at 365 nm | ~165 nm | R7 |
| MCN-8 | Melon | KCl/LiCl | Pt, CoO_x_ | 13.1% at 365 nm | ~713 nm | This work |

**Table S5.** Physicochemical properties of the samples.

| **Sample** | SA^[a]^  [m^2^ g^-1^] | PV^[b]^  [cm^3^ g^-1^] | PD^[c]^  [nm] |
| --- | --- | --- | --- |
| MCN-8 | 11 | 0.04 | 10 |
| PTI-A-24 | 17 | 0.13 | 18 |
| PTI-B-24 | 27 | 0.12 | 12 |

[a] BET surface area. [b] Pore volume. [c] Average pore size determined by the BJH method.

**S3 References**

[S1] L. Lin, C. Wang, W. Ren, H. Ou, Y. Zhang, X. Wang, *Chem. Sci.* **2017**, 8, 5506.

[S2] L. Lin, Z. Lin, J. Zhang, X. Cai, W. Lin, Z. Yu, X. Wang, *Nat. Catal.* **2020**, 3, 649.

[S3] M. Liu, C. Wei, H. Zhuzhang, J. Zhou, Z. Pan, W. Lin, Z. Yu, G. Zhang, X. Wang, *Angew. Chem. Int. Ed.* **2022**, 61, e202113389.

[S4] M. Liu, G. Zhang, X. Liang, Z. Pan, D. Zheng, S. Wang, Z. Yu, Y. Hou, X. Wang, *Angew. Chem. Int. Ed.* **2023**, 62, e202304694.

[S5] J. Wang, Z. Q. Huang, L. Nie, *ACS Nano* **2024**, 18, 26902.

[S6] G. Zou, Q. Wang, G. Ye, Z. Pan, S. Wang, M. Anpo, G. Zhang, *Adv. Funct. Mater.* **2025**, 2420899.

[S7] Q. Wang, G. Zhang, W. Xing, Z. Pan, D. Zheng, S. Wang, Y. Hou, X. Wang, *Angew. Chem. Int. Ed.* **2023**, 62, e202307930.
